# A whole-body micro-CT scan library that captures the skeletal diversity of Lake Malawi cichlid fishes

**DOI:** 10.1038/s41597-024-03687-1

**Published:** 2024-09-10

**Authors:** Callum V. Bucklow, Martin J. Genner, George F. Turner, James Maclaine, Roger Benson, Berta Verd

**Affiliations:** 1 University of Oxford, Department of Biology, OX1 3SZ Oxford, United Kingdom; 2 University of Oxford, Department of Earth Sciences, OX1 3AN Oxford, United Kingdom; 3 University of Bristol, School of Biological Sciences, Bristol, BS8 1TQ United Kingdom; 4 Bangor University, School of Natural Sciences, Bangor, LL57 2UR United Kingdom; 5 Natural History Museum, London, SW7 5BD United Kingdom; 6 American Museum of Natural History, New York City, NY 10024 USA

**Keywords:** Adaptive radiation, Taxonomy

## Abstract

Here we describe a dataset of freely available, readily processed, whole-body *μ*CT-scans of 56 species (116 specimens) of Lake Malawi cichlid fishes that captures a considerable majority of the morphological variation present in this remarkable adaptive radiation. We contextualise the scanned specimens within a discussion of their respective ecomorphological groupings and suggest possible macroevolutionary studies that could be conducted with these data. In addition, we describe a methodology to efficiently *μ*CT-scan (on average) 23 specimens per hour, limiting scanning time and alleviating the financial cost whilst maintaining high resolution. We demonstrate the utility of this method by reconstructing 3D models of multiple bones from multiple specimens within the dataset. We hope this dataset will enable further morphological study of this fascinating system and permit wider-scale comparisons with other cichlid adaptive radiations.

## Background & Summary

Cichlids are one of the most speciose families of vertebrates, with over 1000 species in the African Rift Valley alone^1,2^. Multiple, independent, adaptive radiations of these fishes have evolved in the Great Lakes of East Africa, their associated satellite water bodies, as well as their connecting riverine systems. The radiations of these fishes (Subfamily: Pseudocrenilabrinae^3^), particularly those associated with Lakes Malawi, Victoria and Tanganyika, have become powerful models for the study of macroevolutionary processes^4–10^, behaviour and physiology^11–15^, and have emerged more recently as models in evolutionary developmental biology^16–19^.

Lake Malawi haplochromine cichlids represent a particularly speciose and phenotypically diverse adaptive radiation of lacustrine fishes. This diversity, comprising approximately 850 species of maternal mouthbrooders, is the most extensive adaptive radiation of vertebrates so far identified^1,9^. Molecular clock analyses estimate the radiation to be approximately 800 thousand years old^4^, a relatively young radiation when compared to the older system of Lake Tanganyika (~10myr) which contains just 250 species^7,20^. Despite their high phenotypic diversity, genetic variation between Lake Malawi cichlids is extremely low. Whole genomic comparisons of representatives from all seven distinct ecomorphological groups within Lake Malawi, estimated an average DNA sequence divergence of just 0.19–0.27%^4^ - a range comparable to that within human populations^6^. In addition, a relatively low DNA mutation rate; that alone cannot account for the estimated divergence time of Lake Malawi cichlids^4,6^ and overlapping distributions of inter- and intraspecific (heterozygosity) genetic variation^4^ only further complicates this enigmatic adaptive radiation.

East African cichlids, including those belonging to the Lake Malawi radiation, have recently emerged as powerful models in evolutionary developmental biology^16–19^. Evolutionary modification of embryological mechanisms drives the evolution of novel adaptations and requires genetic variation^21^. Thus, comparing the embryological development of cichlids, which have limited genetic variation, can enable us to identify specific cases where evolution has modified developmental mechanisms^17^. The diversity of feeding habits of Lake Malawi cichlids, and the ability to causally link morphological differences in craniofacial morphology to these ecological niches, has enabled integrative genetic and morphological studies examining the evolution of these traits^22–25^. More recent studies have expanded the scope beyond craniofacial phenotypes, including pigmentation patterning^26–28^; body and fin shape^19,29^ and axial elongation^18^. In parallel, aided by developments in whole-genome sequencing technologies^6^, it has been possible to considerably improve our understanding of the phylogenetic relationships among Lake Malawi cichlids^4,9,30,31^. Previously intractable macroevolutionary studies, such as the convergent evolution of hypertrophied lips^32^ can now take advantage of relatively robust phylogenies based on whole-genome sequences. Moreover, there are now opportunities to use this new phylogenetic information to focus on the evolution of other traits, such as the axial and appendicular skeleton, that is of key importance in teleost diversification^33,34^. However, a whole-body *μ*CT-scan dataset of Lake Malawi cichlid fishes that captures the skeletal diversity present in the adaptive radiation has not yet been described.

**Fig. 1:**
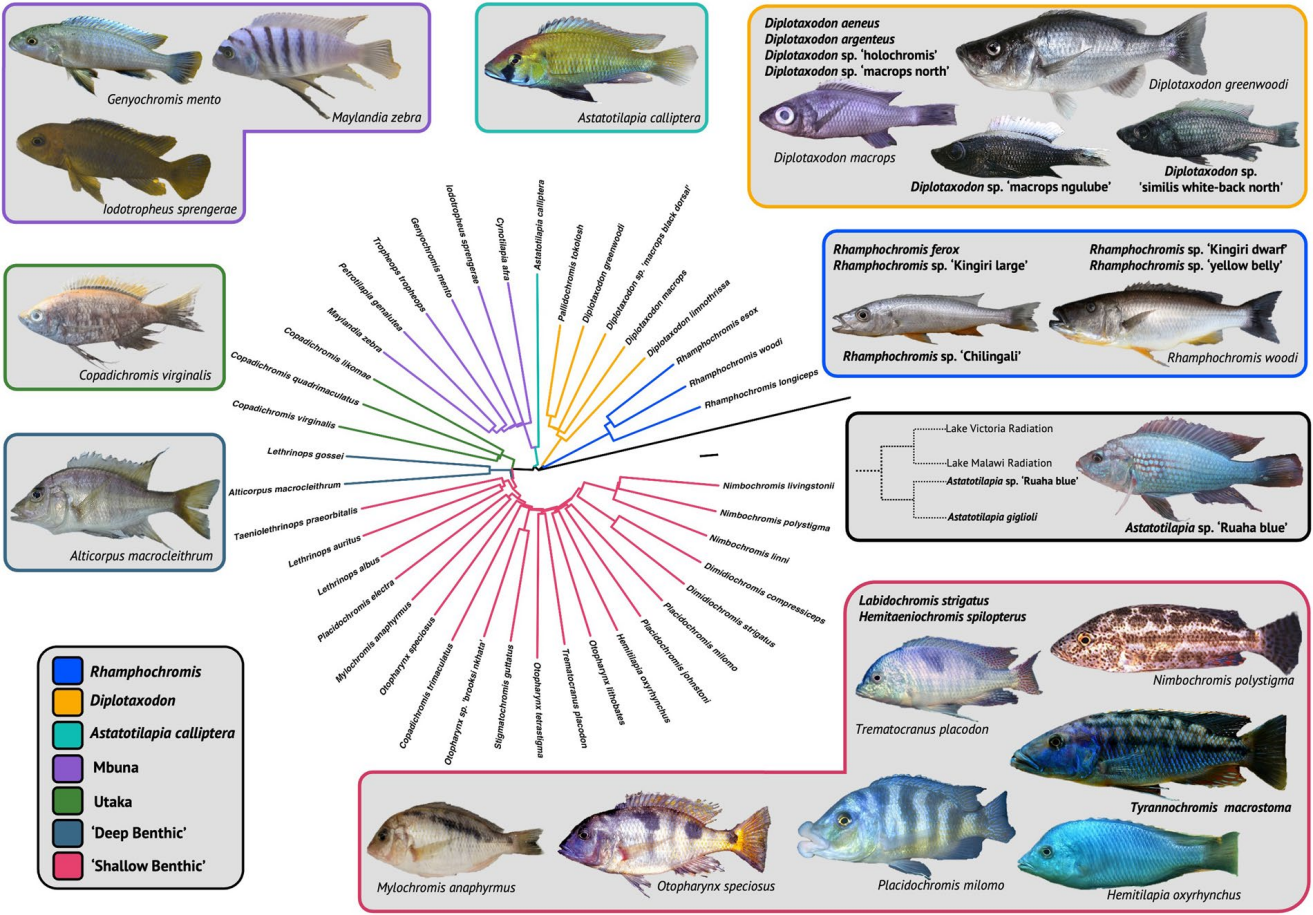
A summary of the *μ*CT-scan dataset. We were able to sample species from all seven ecomorphological groups in the Lake Malawi haplochromine radiation. The phylogenetic relationships between the majority of the species scanned is indicated and coloured according to the respective ecomorphology. The tree is a pruned version of the full (no intermediates) neighbour-joining tree published by Malinsky *et al*.^4^, which is rooted to *Neolamprologous brichardi*, a non-haplochromine cichlid endemic to Lake Tanganyika^20^. Longer terminal branches reflect a higher ratio of within-species to between-species variation. A cladogram depicting the relationship between the Lake Victoria, Lake Malawi and the *Astatotilapia* species native to the Great Ruaha River is indicated in the black box. We also scanned 18 species of cichlid whose phylogenetic relationships are not resolved in the phylogeny shown. The names of these species, most of which are undescribed, are indicated in their respective ecomorphological group in bold. Pictures (not to scale) of example species belonging to each ecomorphological group are also shown. Black bar: 2 × 10^−4^ substitutions per base pair. Fish images used with permission from Ad Konings (*Alticorpus macrocleithrum*, *Diplotaxodon greenwoodi*, *Genyochromis mento*, *Hemitilapia oxyrhynchus*, *Iodotrophesus sprengerae*, *Nimbochromis polystigma*, *Placidochromis milomo* and *Trematocranus placodon*), George F. Turner (*Astatotilapia sp*. ‘Ruaha blue’^183^, *Diplotaxodon macrops*, *Mylochromis anaphyrmus*, *Otopharynx speciosus* and *Rhamphochromis woodi*), Martin J. Genner (*Diplotaxodon sp*. ‘similis white-back north’, *Diplotaxodon sp*. ‘macrops ngulube’ and *Rhamphochromis sp*. ‘Chilingali’), Hannes Svardal (*Copadichromis virginalis*) and Callum V. Bucklow (*Maylandia zebra*). Fish images are not to scale.

Here we present a new database of high-resolution X-ray micro-computed tomography (*μ*CT) scans of Lake Malawi cichlids, providing 3D data on skeletal morphology for the whole body of 56 species across 26 genera. In total these data comprise 116 individuals (56 species, 26 genera) from seven recognized ecomorphological groupings^4^ (Fig. 1, Table 1), contrasting in multiple aspects of morphology, size, behaviour, and habitat preference. We demonstrate the resolution and utility of our dataset by illustrating 3D whole-body renderings of several species, and of several skeletal regions of interest. Our dataset now joins two other East African adaptive radiation datasets, including the recent haplochromine Lake Victoria library^35^ and extensive *μ*CT-scan dataset of Lake Tanganyika cichlid fishes^7^, permitting the examination of macroevolutionary patterns common to adaptive radiations^36,37^ and the macroevolutionary dynamics of convergent evolution. We also describe a methodology to efficiently *μ*CT-scan multiple specimens simultaneously; reducing scanning time and financial cost, whilst maintaining scan quality and demonstrate the utility of this method by reconstructing 3D-models of multiple bones from multiple specimens within our dataset. We hope the availability of these data will inspire others to address some of the many questions still left to understand this remarkable adaptive radiation, permit wider-scale comparisons with other cichlid adaptive radiations, and set a precedent to make whole-body *μ*CT scans the automatic standard for any sampling efforts involving cichlids.

**Table 1:** Genera and species represented within the dataset.

Ecomorphology	Genera Sampled	Genera Sampled (%)	Species Sampled	Species Sampled (%)
*Astatotilapia (calliptera)*	1 of 1	100%	3*	N/A
Deep Benthic	2 of 5	40%	3 (5^†^) of 150	2.00% (3.33%)
*Diplotaxodon*	2 of 2^‡^	100%	9 of 19	47.37%
Mbuna	7 of 12	58.33%	7 of 328	2.13%
*Rhamphochromis*	1 of 1	100%	10 of 14	71.43%
Shallow Benthic	12 of 32	37.50%	20 of 287	6.97%
Utaka	1 of 1	100%	4^§^of 55	7.27%
**Total**	**26 of 54**	**48.15%**	**56 of 856**	**6.54%**

The number of genera and species for each ecomorphological group are the same as those used in Malinsky *et al*.^4^. *Astatotilapia calliptera, *Astatotilapia* sp. ‘Ruaha blue’ and *Astatotilapia gigliolli*. ^†^If considering the addition of *Lethrinops albus* and *Lethrinops auritus* that cluster within the ‘shallow benthics’. ^‡^Includes *Pallidochromis*. ^*§*^Includes *Copadichromis trimaculatus* which clusters within the shallow benthics in the phylogeny depicted in Fig. 1.

## Methods

### Sample Selection

There are an estimated 850 species of Lake Malawi cichlid fishes^4^. Many of which have not been described, preserved in museum collections or are available on phylogenies based on whole genome evidence. Therefore, to maximise the utility and morphological variation captured by our dataset, we focused on species present in published phylogenies^4,9,24^, and sought to include as many genera as possible. Moreover, we prioritised scanning the type species for genera, and avoided inclusion of species which already had whole body scans available on Morphosource. We were able to sample 26 of the 53 (49.05%) endemic genera listed on the curated species list on malawi.si^38^, or 48.15% of the 54 genera previously cited^4^ (see Table 1). Our specimens were sourced from the collections at the Natural History Museum in London (NHMUK), from the School of Biological Sciences of the University of Bristol (Martin J. Genner) and from the School of Natural Sciences of Bangor University (George F. Turner). In total we scanned 116 specimens from 56 species (Supplementary Table S1). Of these, 99 were wild-caught and 17 were laboratory-reared. Laboratory-reared specimens included *Astatotilapia calliptera* (Mbaka River, n=10), *Maylandia zebra* (Boadzulu island, n=5) and *Rhamphochromis* sp. ‘Chilingali’ (n=2), all of which died naturally or were compassionately euthanised by anaesthetic overdose [Schedule 1; Animals (Scientific Procedures) Act 1986]. All animal work was conducted following approval by the Departmental Animal Welfare Ethical Review Body (AWERB) for the Department of Biology at the University of Oxford.

### *μ*CT-Scanning

**Fig. 2:**
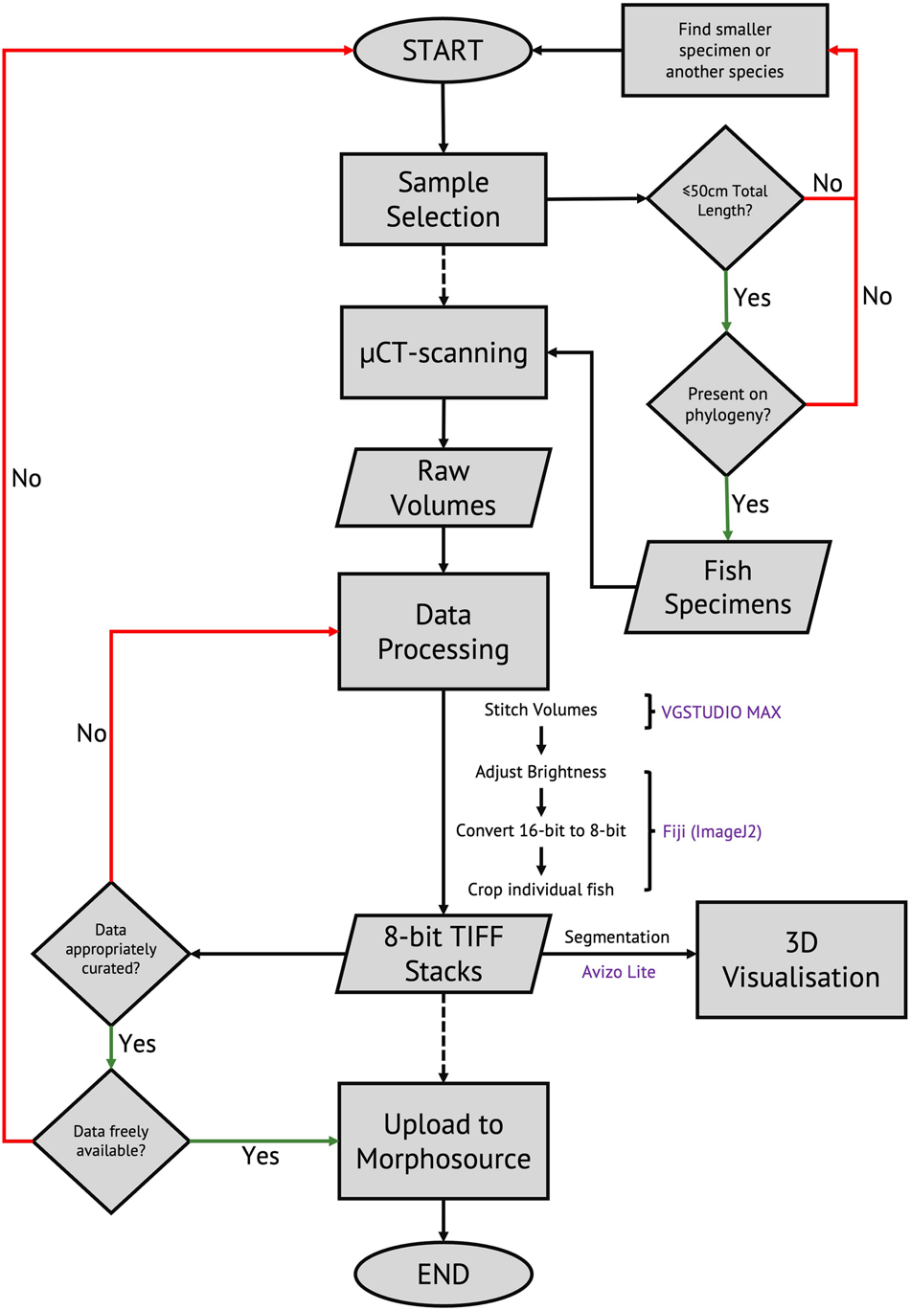
Flowchart of *μ*CT-scanning, image processing and segmentation methodology. The flowchart outlines the necessary decisions that were made during collation of the described *μ*CT scan dataset. Rectangles represent processes; parallelograms represent inputs or outputs; diamonds represent decisions. It is sufficiently generalised that it can be reused for future data collection. We were focused on generating data for a specific macroevolutionary study, so we restricted the dataset to species with known phylogenetic placements but this is not strictly necessary. Software associated with data processing steps are indicated in purple. Further information about processing and segmentation is provided in the Usage Notes.

A flowchart describing all the necessary decisions and required processing steps is provided in Fig. 2. Since there was already a large collection of specimens present at the Natural History Museum in London (NHMUK) and in the extensive research collections of Martin J Genner (School of Biological Sciences, University of Bristol) and George F Turner (School of Natural Sciences, Bangor University) we decided to take advantage of the scanners present in the CT facility of NHMUK and at the XTM Facility based in the Paleobiology Research Group at the University of Bristol, respectively. Of the total 116 individuals scanned (56 species), 56 specimens (28 species) were scanned at the NHMUK Imaging and Analysis Centre and 60 specimens (28 species) were scanned at the XTM Facility at the University of Bristol.

#### Scanning Arrangement

**Fig. 3:**
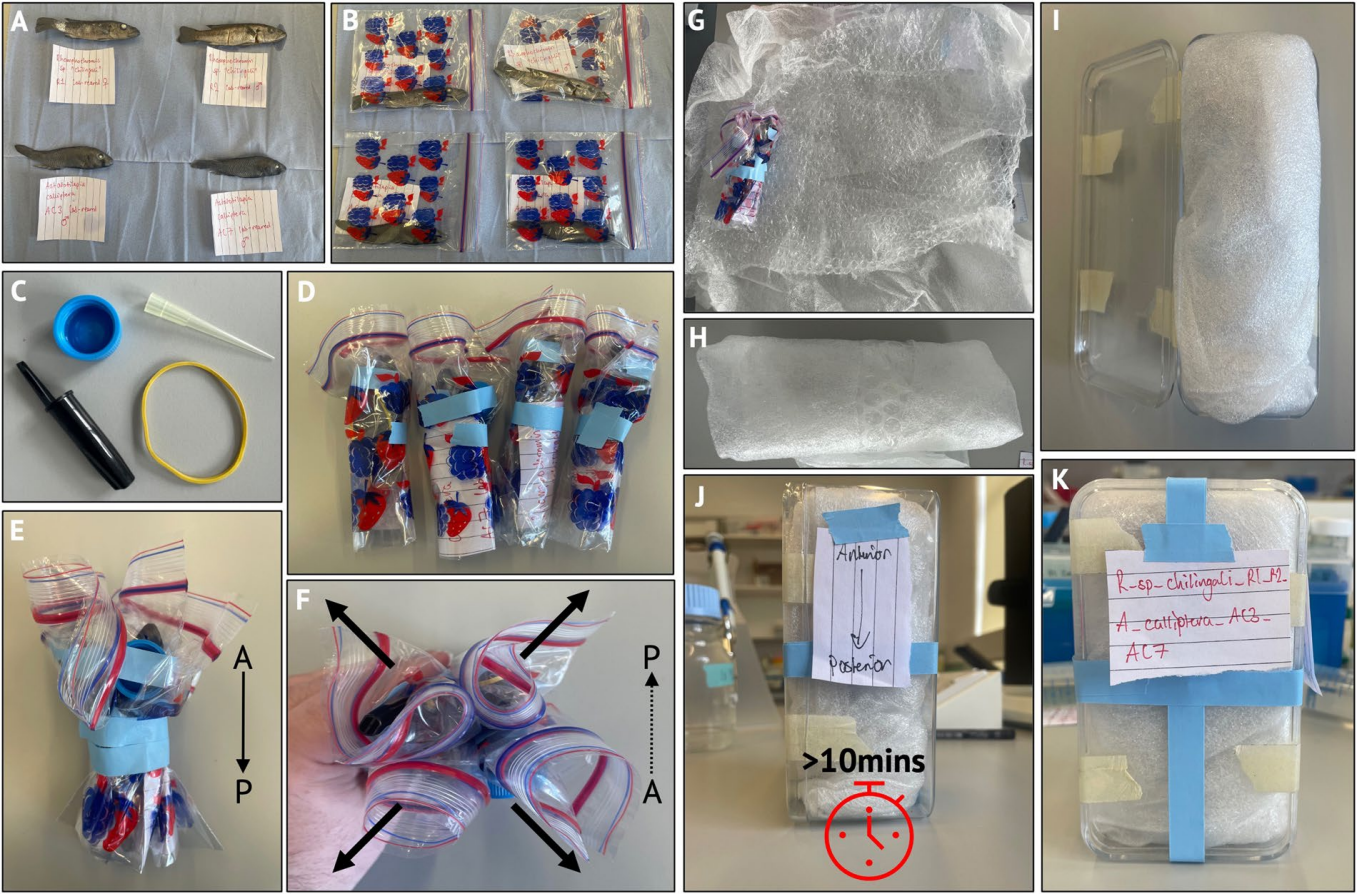
Specimen preparation for *μ*CT-scanning. Multiple fish were scanned at the same time (**A**). Individual fish were labelled and placed in separate plastic bags so they could be correctly identified and stored after imaging (**B**). Unique objects (**C**) that would be readily identifiable following *μ*CT-scanning were attached to the outside of these bags, ideally close to the heads, positioned outwards (**F**, arrows), and bundled together with tape (**D**–**F**) all with the same orientation (head-up). Bundles were then wrapped in bubble wrap and other packaging material (**G,H**) and tightly sealed inside a plastic container, again head-up (**I**). Containers were left for at least ten minutes to settle to prevent movement during scanning (**J**) and an additional label was placed on the container to permit future identification if multiple batches were prepared together (**K**).

To maximise the utility of our time and the number of species scanned, multiple specimens were scanned in each individual scan (Fig. 3). Each batch of specimens was fit to the width of the scan field of view to maximise resolution, and multiple scans were conducted along a the vertical axis in order to scan the full body length of each specimen. Therefore, batches of similarly sized fish were packaged together prior to scanning to ensure multiple individuals could be simultaneously captured within the field of view during scanning. Batch sizes varied between two and five specimens, with the number of each batch ultimately dependent upon the overall size of the specimens within the batch. Of the 32 batches scanned: 20 were comprised of four specimens; nine of three specimens, and two and one batch(es) of one and five specimens, respectively. Since multiple individuals of different species were often scanned together (Fig. 3A,B) it was critical that individuals of the same species could be readily identified. Therefore, unique, low density objects such as plastic bricks, pipette tips and rubber bands were placed in physical proximity to each specimen, to act as recognisable markers (Fig. 3C) which would readily resolve in the reconstructed image stacks (see Post-Scanning Processing). These objects were attached to each individual specimen-containing bag, and the specimens were bundled together, ideally ensuring that the objects faced outwards (Fig. 3D–F). Specimens were tightly packed into plastic containers, sealed with tape, and allowed to rest upright (head-up) for at least ten minutes so the contents could settle to prevent movement during scanning (Fig. 3G–K). We were able to scan, on average, 23 specimens per hour at maximal efficiency, an efficiency that was primarily the result of having two people per scanning visit (one scanning and one packing). The scanning rate could be further increased by packaging specimens in advance of the scanning so that subsequent batches can be scanned with no delay.

#### Scanning Procedure

Prior to scanning, a visual inspection of the specimens was made following X-ray exposure to check for internal damage (such as damage to the vertebral column). If present, and possible, specimens were switched for another specimen of the same collection. To maximise scan resolution, each batch of fish was scanned using the full width of the scanner field of view. This necessitates that multiple, overlapping scans were conducted along the vertical axis of the scanner (Z-axis) in order to capture the full body length of each fish. The overlapping scans were subsequently stitched together in the processing stage using the software, VGStudio Max 3.2.5 64-bit (see below). The fish scanned ranged in standard length of 3.8cm to 33cm, which meant that scanning parameters varied between batches. The number of projections generated range from 901 to 2001. Exposure times varied between 250 and 500 seconds, with a median of 354 seconds. Similarly, power varied between scans, ranging between 12.480 and 37.995 watts (W) (mode 37.440W). All scanning parameters, for each batch conducted, as well as for each individual scan can be found in the metadata supplied in Supplementary Table S1.

### Post-Scanning Processing

All processing and segmentation was conducted on a machine specifically built for image analysis, with the following specifications: 2 x Intel® Xeon® CPU-ES-2640 @2.60GHz, 2601MHz, 8 Core(s) processors, 128GB of dedicated DDR3 RAM running on Microsoft Windows 10 Pro (Build Number: 10.0.19045). Raw, isometric volumes generated from the CT-scanning were imported into VGStudio Max 3.2.5 64-bit and anterior and posterior halves were subsequently stitched together by defining overlapping regions of interest in both the anterior, middle (if applicable) and posterior volumes. 16-bit tiff stacks were exported from VGStudio Max 3.2.5 64-bit and imported into FIJI^39^, a GUI for ImageJ^40^. In FIJI, individual fish were cropped out of the 16-bit stacks, which were identifiable due to the unique objects associated with each individual (see above). The brightness was adjusted by extending the distribution of pixel values to remove 0 values, and the tiff stacks were converted into 8-bit to decrease file size. Total stack file size was further decreased by removing images at the beginning and end of the exported tiff stacks that did not contain readily identifiable bone or tissue.

### Segmentation and Visualisation

Reconstructed image stacks were imported into Avizo Lite (v9.3.0), a proprietary software developed by Thermo Fisher Scientific and generated either a full body volume render using the volume generation tool or manually rendered 3D models of the whole body following a rough manual segmentation of the whole body. Optimal threshold values were manually chosen based on the volume or 3D rendering and all whole-body 3D models were smoothed with a smoothing factor of 2.5. Surfaces from 3D whole-body renderings were exported from AvizoLite as Polygon File Format (.ply) files and imported into MeshLab for visualisation and manipulation. See the Usage Notes for further notes on segmenting the *μ*CT-scans in the dataset.

## Data Records

All data is freely avaliable and has been deposited into a dedicated project on Morphosource^41^, which includes cropped 8-bit tiff stacks of 116 specimens^42–157^, representing 56 species. Coverage of the Lake Malawi radiation by our dataset^41^ is outlined in Table 1. The names of all 56 species present in the dataset, including their respective ecomorphological group, is listed in Fig. 1. Full specimen details, including scanning parameters, can be found in Supplementary Table S1. Detailed discussion of the species in the dataset, including suggestions for possible macroevolutionary and systematic studies can be found in the Usage Notes (see below).

## Technical Validation

**Fig. 4:**
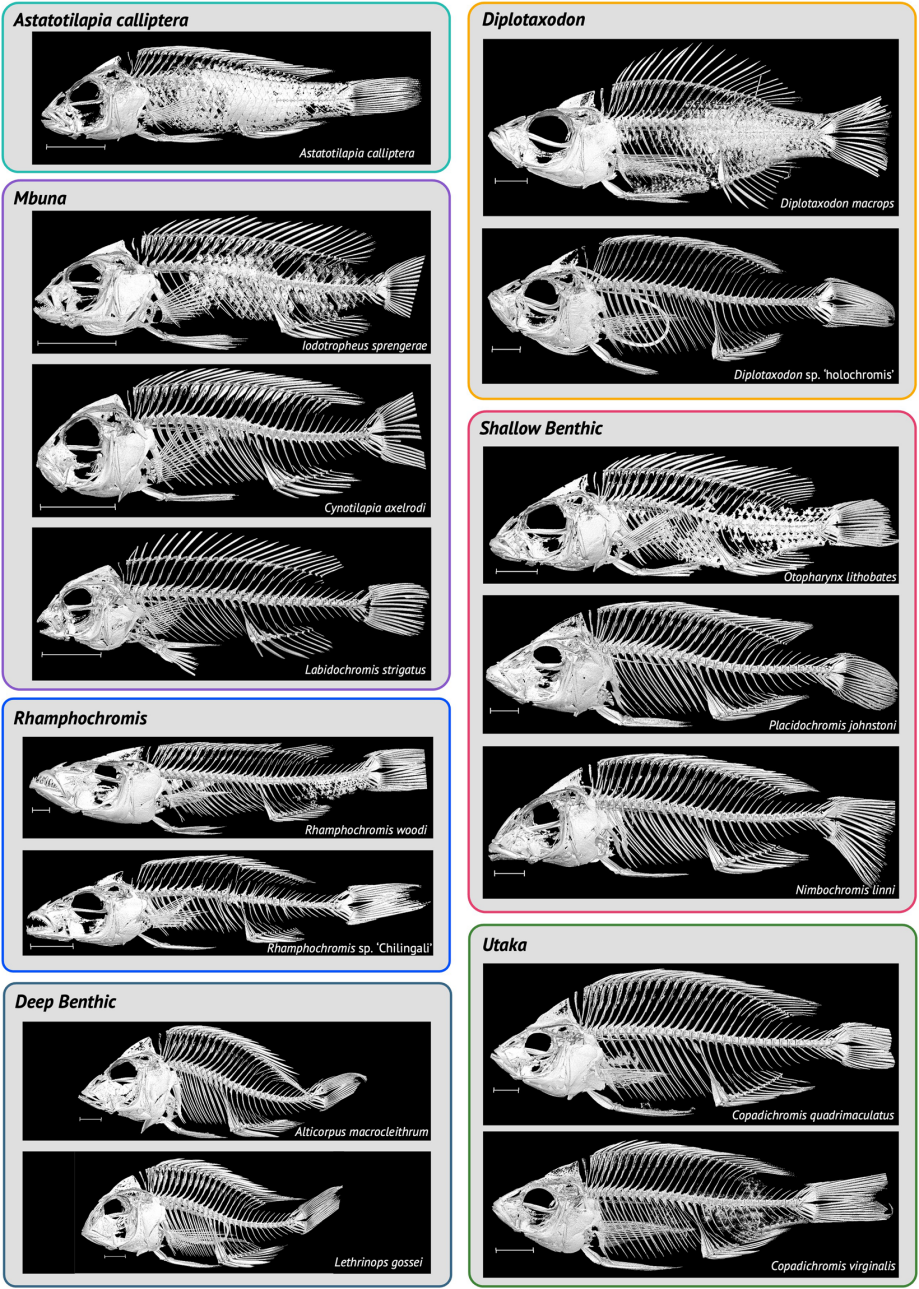
Whole-body 3D models of select specimens from the dataset. Specimens are arranged according to the ecomorphological group they belong to. Species names are indicated. The ring structure in *Diplotaxodon* sp. ‘holochromis’ and *Lethrinops gossei* is a rubber band used for identification purposes. Scale for all images is shown as 1cm. See Supplementary Table S1 for details of the specimens used.

**Fig. 5:**
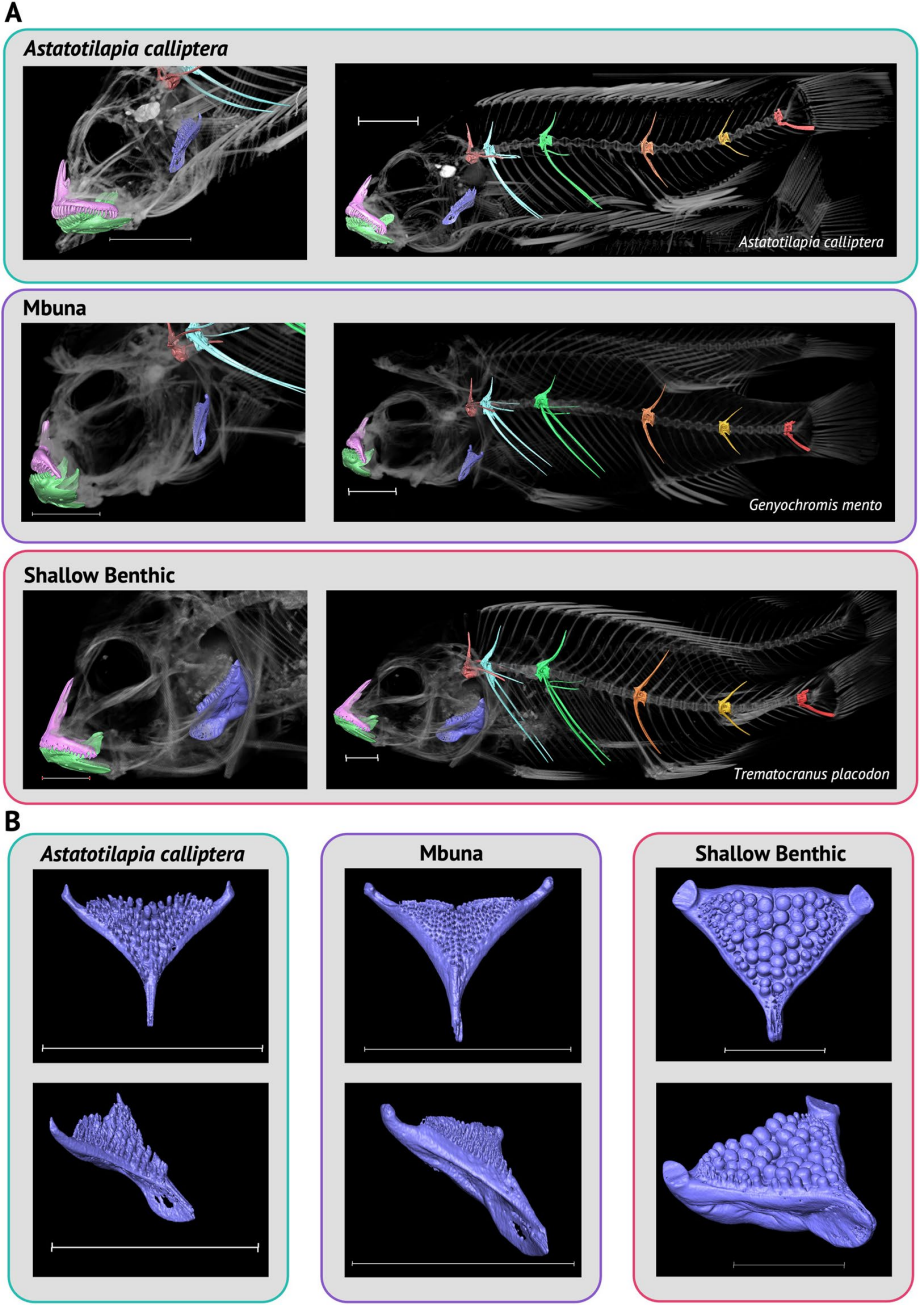
Segmented Bones from *Astatotilapia calliptera*, *Genyochromis mento* (mbuna) and *Trematocranus placodon* (shallow benthic). (**A**, left) A close up, lateral view of the head of each species (species name indicated on right), showing the dentary (green), premaxilla (pink) and lower pharyngeal jaw (purple) positioned within a volume render of the head. (**A**, right) A whole body lateral view showing the aforementioned jaw bones, as well as the first non-rib-bearing vertebra (orange), the first rib-bearing (precaudal, PC) vertebrae (light blue), PC8 (green), non-rib bearing (caudal, CV), CV3 (orange), CV10 (gold) and the pre-urostyle vertebrae (red). (**B**) Anterior (top) and anterolateral (bottom) view of the lower pharyngeal jaws for each species in (A). Scale for all images is 1cm. See Supplementary Table S1 for details of the specimens used. 3D models for all segmented bones can be found in the Supplementary Material.

**Fig. 6:**
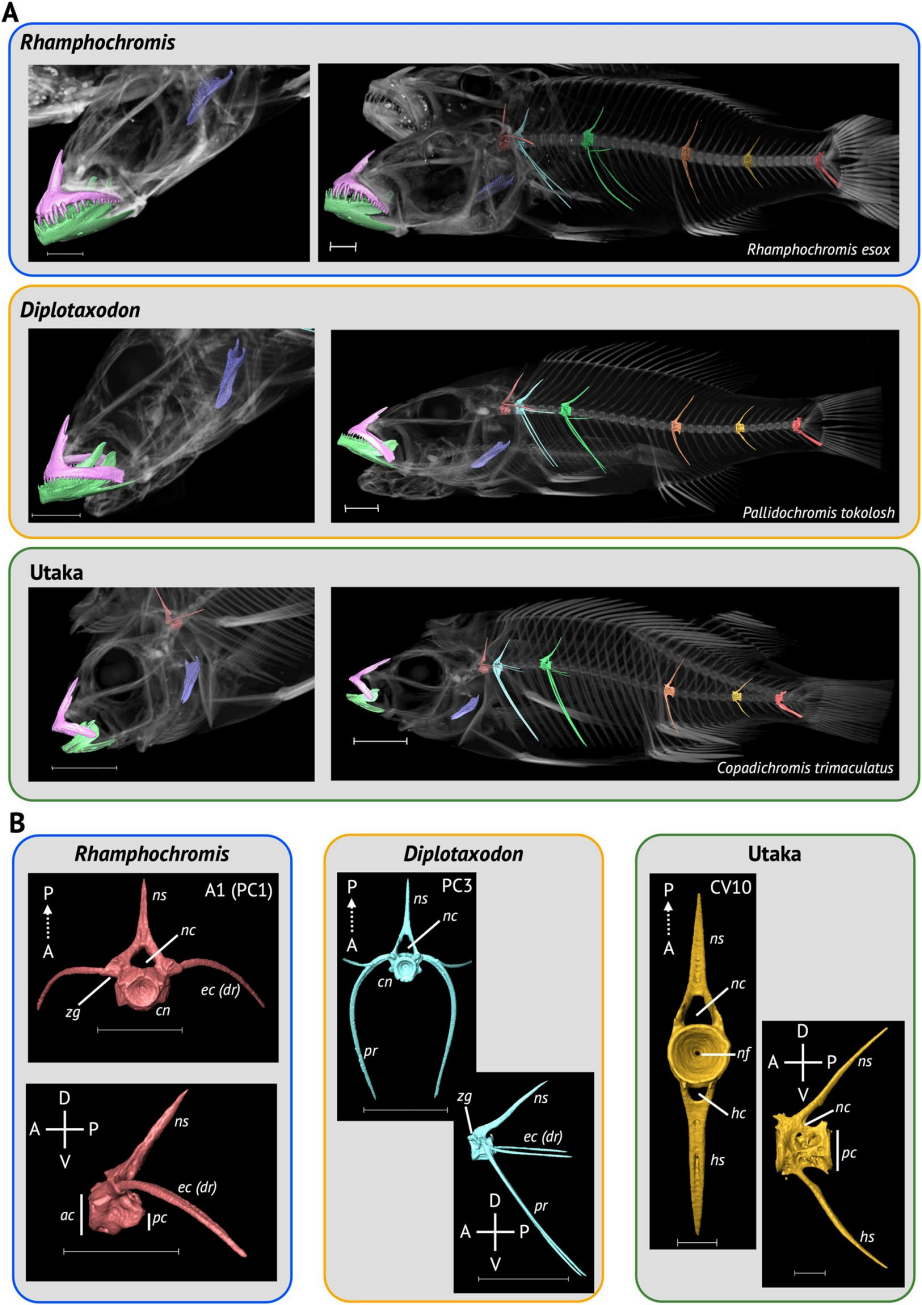
Segmented Bones from *Rhamphochromis esox* (*Rhamphochromis*), *Pallidochromis tokolosh* (*Diplotaxodon*) and *Copadichromis trimaculatus* (Utaka). (**A**) Left, lateral view of the head of each species, showing the dentary (green), premaxilla (pink) and lower pharyngeal jaw (purple). Right, whole body lateral views showing aforementioned jaw bones, as well as the first non-rib-bearing vertebrae (orange), the first rib-bearing (precaudal, PC) vertebrae (light blue), PC8 (green), non-rib bearing (caudal, CV), CV3 (orange), CV10 (gold) and the pre-urostyle vertebrae (red). (**B**) Images of select vertebrae (indicated) from each species shown in (A). Axes are indicated (A, anterior; P, posterior; D, dorsal; V, ventral). Vertebral models are labelled (*ac*, anterior cone span; *cn*, centrum; *ec* (*dr*), epicentrals (dorsal ribs); *hc*, haemal canal; *hs*, haemal spine; *nc*, neural canal; *nf*, neural foramen; *ns*, neural spine; *pc*, posterior cone span; *pr*, pleural ribs; *zg*, zygapophyses). Scale for all images is 1cm, besides CV10 for Utaka which is 0.1cm. See Supplementary Table S1 for details of the specimens used. 3D models for all segmented bones can be found in the supplementary material.

Example whole-body, scaled, 3D renderings of representatives from each ecomorphological group can be found in Fig. 4 and for every specimen (mix of volume and model renders) in the dataset in the Supplementary Material. To demonstrate the quality of 3D-models that can be segmented from specimens in our dataset we manually segmented multiple bones, including the dentary, premaxilla, lower pharyngeal jaw and multiple vertebral types in several species discussed below (see Usage Notes). This included: the ‘generalist’ *Astatotilapia calliptera*; the ‘mbuna’ and lepidophage (scale-eater), *Genyochromis mento*; the ‘shallow benthic’ and snail crusher, *Trematocranus placodon* (Fig. 5); the pelagic piscivores, *Rhamphochromis esox* and *Pallidochromis tokolosh* and the zooplanktivorous (utaka), *Copadichromis trimaculatus* (Fig. 6). Lower pharyngeal jaws that are highly variable among species of Lake Malawi cichlids^25^ were particularly well resolved. For example, newly erupting teeth were visible on the relatively large, and dense, lower pharyngeal jaw of *Trematocranus placodon* (Fig. 5B). Similarly, renderings of multiple vertebral types (Figs. 5–6) were also of good quality. The zygapophyses and fine structure of the vertebral centra, sometimes including the neural foramen, were also well resolved. All 3D-renderings of these bones can be found in the Supplementary Materials as downloadable .ply files.

**Fig. 7:**
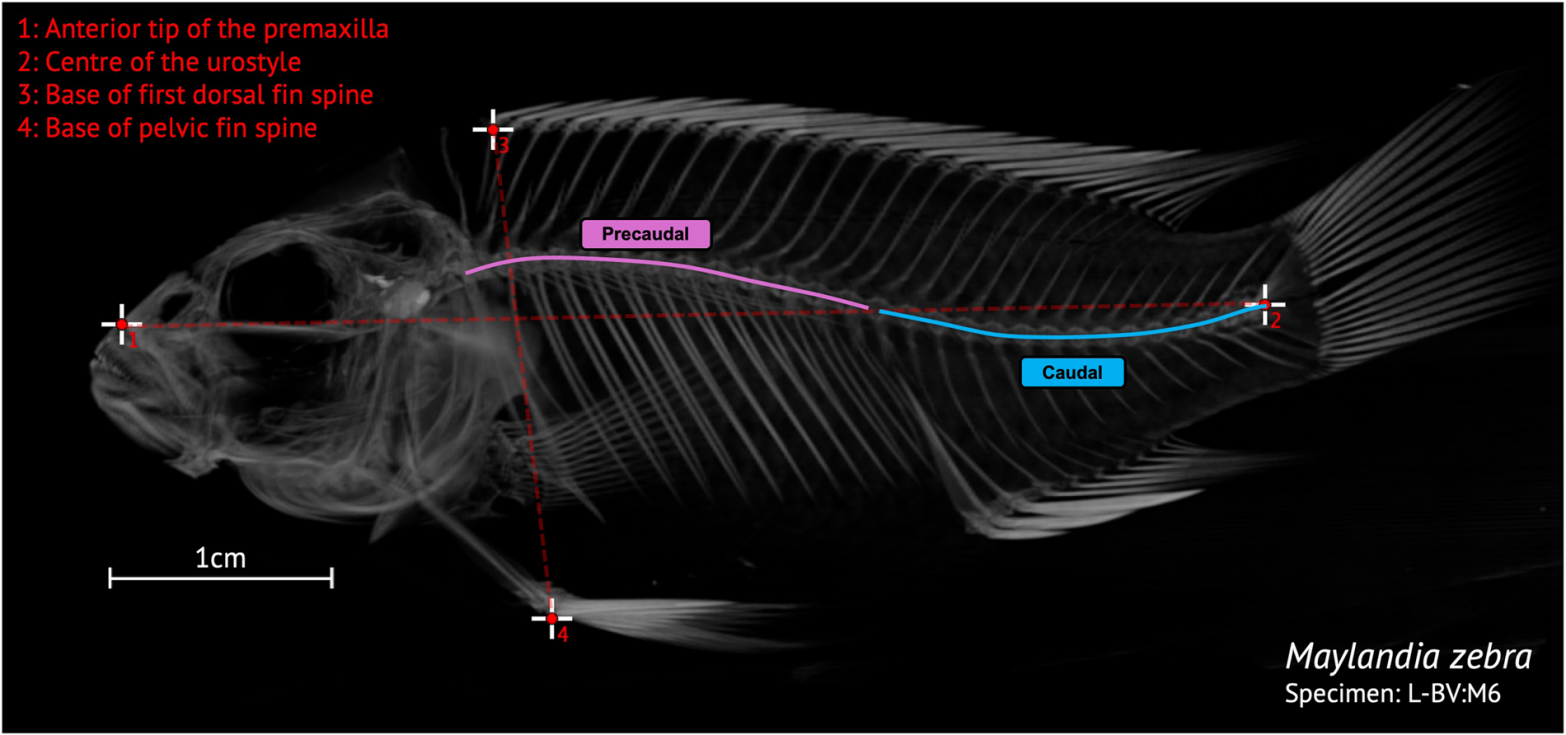
Example specimen volume rendering with body aspect ratio landmarks. The number of precaudal (pink) and caudal vertebrae (blue), including the urostyle, the total number of vertebrae (sum of the precaudal and caudal vertebrae, including the urostyle) and the body aspect ratio was estimated for 113 of the 116 specimens in the dataset. The landmarks used to estimate the body aspect ratio are indicated in the figure on an example specimen (*Maylandia zebra*, L-BV:M6, see Supplementary Table S1). Landmarks 1 and 2 were used to calculate the length, and landmarks 3 and 4 the width by calculating the length of a straight line between the x,y coordinates. Landmark 1, anterior tip of the premaxilla; Landmark 2, centre of the urostyle; Landmark 3, Base of the first dorsal fin spine; Landmark 4, base of the pelvic fin spine.

In addition, to demonstrate that our data will also be useful for the collection of meristic data, we also counted the number of precaudal, caudal and total number of vertebrae (including the urostyle), as well as an estimation of the body aspect ratio for 113 of the 116 specimens in Supplementary Table S2. The three remaining specimens were deformed or poorly rendered and vertebral counts or body aspect ratios could not be taken. Precaudal, caudal and total vertebral counts as all as an estimation of the body aspect ratio were estimated from 2D lateral images of either a volume rendering or 3D-model of the whole body of the specimen (Fig. 7). Lateral images of a volume or 3D whole body rendering can be found in the Supplementary Material for every specimen in the dataset. Length landmarks were placed on the anterior tip of the premaxilla and in the centre of the urostyle and width landmarks were placed at the base of the dorsal fin spine and pelvic fin spine, respectively (see Fig. 7). We note that these data could also be collected with the use of 2D radiographs. However, *μ*CT-scanning adds the additional possibility of also generating 3D models which can be the basis of studies that consider and compare overall, complex bone shape that would otherwise be missed (3D geometric morphometrics), or undetectable with the use of 2D radiographs. Therefore, the *μ*CT-scans in our dataset provide can both provide well resolved models for geometric morphometric studies comparing complex bone shape between Lake Malawi cichlids as well as a useful source of meristic data for macroevolutionary studies. We have included suggestions of possible macroevolutionary studies that could be conducted with these data in the Usage Notes.

## Usage Notes

### *Diplotaxodon* and *Rhamphochromis*

Nine species of the *Diplotaxodon* group (47%; Table 1), including the type species *Diplotaxodon argenteus* (n=1) and *Pallidochromis tokolosh* (n=2) are present within our dataset^41^. In addition, we sampled 10 species of the *Rhamphochromis* genus (71%; Table 1), including the type species *Rhamphochromis longiceps* (n=2) and the remarkably large *Rhamphochromis woodi* (n=2, see below), that are endemic to Lake Malawi. We were also able to sample two sympatric species from the crater lake, Lake Kingiri, *Rhamphochromis* sp. ‘Kingiri dwarf’ (n=2) and *Rhamphochromis* sp. ‘Kingiri large’ (n=2), as well as *Rhamphochromis* sp. ‘Chilingali’ (n=4) from the satellite, Lake Chilingali (Fig. 4), which is now presumed extinct in the wild^158^.

The *Diplotaxodon* and *Rhamphochromis* groups are two reciprocally monophyletic diverging lineages of Lake Malawi cichlids^4^ that have adapted to the pelagic-limnetic zone of Lake Malawi^159^. The majority of species in the groups are piscivorous, although several species, including *Diplotaxodon limnothrissa* (n=2) are predominantly zooplanktivorous^160^. Large-bodied *Rhamphochromis* primarily feed on Lake Malawi sardines (usipa; *Engraulicypris sardella*) and endemic cichlids (e.g. utaka). Members of *Diplotaxodon* and *Rhamphochromis* are among the deepest-living of all Lake Malawi cichlids, with representatives of both being caught at depths exceeding 200 metres - the ‘twilight zone’ where light is almost completely absent^159^. Species within the *Diplotaxodon macrops* complex, which is represented in the scanned samples by *Diplotaxodon macrops* (n=1) (Fig. 4), *Diplotaxodon* sp. ‘macrops north’ (n=2), *Diplotaxodon* sp. ‘macrops black dorsal’ (n=2) and *Diplotaxodon* sp. ‘macrops ngulube’ (n=2), have been found between 100 and 220m, a depth similarly reported to be occupied by *Rhamphochromis* during the day^161^.

Morphological comparisons of *Diplotaxodon* and *Rhamphochromis* with Lake Malawi cichlids from other habitats could provide valuable insights into convergent adaptation of traits enabling occupation of pelagic niches. Divergence along depth gradients is associated with the evolution of reproductive isolation in many marine and freshwater species groups, likely a consequence of the strong selective pressures associated with deeper water, such as the absence of sunlight, greater hydrostatic pressure, and reduced levels of dissolved oxygen^162,163^. Morphological comparisons of *Diplotaxodon* and *Rhamphochromis*, against closely related littoral species, could be a powerful model for the study of evolution of convergent phenotypes necessary for adapting to pelagic environments. Body elongation, supported by increased vertebral counts, is a adaptive trait common in teleosts adapted to pelagic (and piscivorous) niches^164^, including in *Rhamphochromis*^165^. Moreover, the evolutionary modification of vertebral morphology has been linked to changing swimming kinematics, body shape and habitat preference^166^, including adapting to pelagic environments^34^. Given the remarkable depth preference and rapid divergence of the *Diplotaxodon* and *Rhamphochromis* lineages, a study of body shape, as well as vertebral count and shape could better determine the rate at which these phenotypes can become fixed and provide further insights into the role these morphological adaptations play along the benthic-pelagic speciation axis^162,163^.

Remarkable size variation is present within the *Rhamphochromis* genus. *Rhamphochromis woodi* is considered to be one of the largest Lake Malawi cichlids, measuring a standard length (SL) of up to 40 cm^167^. In contrast, the smallest known member of *Rhamphochromis*, *Rhamphochromis sp. ‘Kingiri dwarf’*, endemic to the crater lake Kingiri, do not exceed 7.5 cm SL in the wild^158^ – a 5.33x length difference just within the same genus. Similarly, wild caught *Rhamphochromis* sp. ‘Chilingali’ are also small bodied, with maximum observed standard length of 10.6 cm^8^, which makes them relatively amenable to laboratory study. Its elongate body, supported by relatively high vertebral counts, has made it a useful model in evolutionary developmental biology^17^, particularly for the study of somitogenesis^18^, the developmental process that gives rise to the vertebral precursors. Nonetheless, given the exceptional size difference in the genus *Rhamphochromis*, our dataset represents a potentially valuable resource for the study of the evolution of allometric scaling, which has not been well studied in cichlids^168^.

### Shallow Benthic

The shallow benthic species group is extremely speciose, comprising hundreds of species that exhibit remarkable morphological^4,169^ diversity. The majority of shallow benthic species inhabit relatively shallow inshore habitats of Lake Malawi, such as the sand or mud lake floor, or sand-rock transitional zones. Our dataset^41^ includes 20 shallow benthic species in 12 genera (Table 1), including several large, ambush predators, as well as a collection of trophic specialists. For a complete list of shallow benthics in our dataset^41^ see Supplementary Table S1.

Large ambush predators represented in the dataset^41^ include *Dimidochromis strigatus* (n=1), *Dimidochromis compressiceps* (n=1), *Tyrannochromis macrostoma* (n=1), *Nimbochromis livingstonii* (n=1) and *Nimbochromis polystigma* (n=2). *Dimidochromis compressiceps* has a generalist piscivore lifestyle, occupying the reed-beds of the Lake. *Nimbochromis livingstonii* and *N. polystigma* are both considered to be ‘sleeper[s]’ (Chichewa: “kaligono”), which bury themselves within the sandy substrate and snatch unsuspecting prey attracted by the disturbed sediment^169^. Another member of *Nimbochromis*, *Nimbochromis linni* (n=1) has a characteristic downward-projecting snout (Fig. 4), enabling it to extract prey from rock crevices^169,170^.

We sampled several shallow-benthic predators, including *Otopharynx speciosus* (n=2), one of the few piscivores within *Otopharynx*. Males of this species have been encountered at depths exceeding 25m^169^, suggesting tolerance of relatively deep water, and suggesting the species may have morphological adaptations enabling occupation of deep-water niches similar to *Rhamphochromis* and *Diplotaxodon*. Of the approximately 20 species of *Otopharynx*^171^ we were able to sample an additional three species: *Otopharynx lithobates* (n=3, including the holotype NHMUK 1974.7.5.1); *Otopharynx tetrastigma* (n=2); and the undescribed *Otopharynx* sp. “brooksi nkhata” (n=1). We also sampled several specialised trophic specialists including the molluscivores *Mylochromis anaphyrmus* (n=1) and *Trematocranus placodon* (n=1) and the invertebrate picker *Placidochromis johnstoni* (n=1, Fig. 4). The diet of *T. placodon* predominately comprises the gastropods *Bulinus nyassanus* and *Melanoides tuberculata*^172^. Enlarged sensory pores and lateral lines form a sonar-like detection system that allows *T. placodon* to sense the movement of these prey within the sediment. Curiously, this strategy and associated morphological characteristics are also associated with *Aulonocara* and *Lethrinops*, both ‘deep benthics’, suggesting convergent evolution of lateral line phenotypes^9^. The specimens in our dataset^41^ may enable morphological comparisons to further investigate differences in sensory pore characteristics among species.

### ‘Rock-dwelling’ *Mbuna*

The mbuna group dominate the rocky shores of Lake Malawi, and are used as a model system for the study of rapid speciation and adaptive radiation^25,173,174^. Similar to the shallow-benthics, there are hundreds of species, many of which are undescribed^169,174^. We aimed to maximise our coverage of the phenotypic diversity in the group by sampling multiple genera, which are largely differentiated on the basis of head, jaw and tooth morphology^174^. Our dataset^41^ includes 7 species (15 individuals) of mbuna, covering 7 of the 14 described mbuna genera (Table 1).

*Cynotilapia* can be distinguished from other genera by the presence of unicuspid (conical) teeth^169,175,176^ and is represented in our dataset by *Cynotilapia axelrodi* (n=1, Fig. 4). This is a genus of typically planktivorous species^169^ and their relatively simple dentition may reflect this lifestyle^177^. By contrast, *Maylandia* (*Metriaclima*^178^), represented by *Maylandia zebra* (n=5), has closely arranged bicuspid teeth, that is uses for pulling and scraping loose Aufwuchs (periphyton) attached to the rocks found in their preferred preferred rocky habitats^175,179^. *Tropheops* and *Iodotropheus*, represented by *Tropheops tropheops* (n=2) and *Iodotropheus sprengerae* (n=2), also have closely packed bicuspid teeth, that they use to feed on epilithic algae which they pluck with sideways, upwards head jerks, a behaviour likely supported by *Tropheops*’ characteristic steeply sloped vomer (71-96^°^)^169^. Members of *Petrotilapia*, represented by *Petrotilapia genalutea* (n=1) have a mixed combination of tricupsid and unicupsid teeth that they use to comb loose peripyton from rock surfaces^180^. A further represented mbuna genus is the monotypic *Genyochromis*, represented by *Genyochromis mento* (n=2). Like the majority of mbuna, *G. mento* has prominent outer bicupsid teeth that are supported by smaller inner tricupsid teeth^169,175^. In contrast to most other mbuna, however, *G. mento* is a highly specialised feeder, a lepidophage (scale-eater), that targets the the caudal and anal fins of other cichlids in rocky habitats^169,175,181^. The preferred striking side of *G. mento* significantly correlates with left-right asymmetry of the dentary, with right and left-leaning individuals preferring to strike the corresponding side, respectively, of their prey. Interestingly, however, a comparison of their jaw laterality with *Perissodus microlepis*, a lepidophage endemic to Lake Tanganyika^20^, showed that laterality in *G. mento* is weaker than in *P. microlepis* – likely a result of phylogenetic constraint from their shorter evolutionary history and their herbivorous ancestors^181^.

The craniofacial bones commonly studied in mbuna, such as the dentary, premaxilla, pharyngeal jaws, as well as their associated teeth, can be segmented from specimens in the dataset (see *G. mento*, Fig. 5) and will be helpful for geometric morphometric analyses focused on examining craniofacial shape differences between mbuna species. Future sampling should focus on the seven remaining genera not sampled in our dataset^41^: *Abactochromis*, *Chindongo*, *Cyathochromis*, *Gepyrochromis*, *Labeotropheus*, *Melanochromis* and *Psuedotropheus*.

### *Astatotilapia calliptera* and Ruaha Catchment

*Astatotilapia* is polyphyletic and current members of the genus are widespread across East and North Africa^6,182,183^. Only one species of *Astatotilapia* is native to Lake Malawi, *Astatotilapia calliptera*, which is also found in East African rivers flowing eastward to the Indian Ocean, from the Rovuma River in the north, to the Save River in the south. Given the wide distribution of the species, it is perhaps unsurprising that intraspecific genetic variation within the species is comparable to that of the whole Lake Malawi radiation^4,6^. Despite their wide distribution and relatively large intraspecific genetic variation, they phylogenetically cluster within the Lake Malawi radiation (Fig. 1), forming a sister clade to the mbuna, with which they share an excess of alleles^4^. This pattern, alongside a perceived riverine ‘generalist’ lifestyle, has led to the hypothesis that either Lake Malawi cichlids radiated from an *A. calliptera*-*like* ancestor or that *A. calliptera* is the sympatric ancestor of all Lake Malawi cichlids^4,6,169,183^.

Given the importance of *A. calliptera* in the Lake Malawi radiation, we sampled multiple individuals from multiple populations. We scanned nine laboratory-reared individuals from the Mbaka river population, which flows into the northern end of Lake Malawi^184^. We also scanned individuals from Lake Chilwa (an endorheic lake south-east of Lake Malawi^185^; n=2), Lake ‘Misoko’, presumably Lake Masoko (a crater lake north of Lake Malawi^184^, n=2), and wild-caught individuals from the main body of Lake Malawi (n=2). Populations of *A. calliptera* differ in life history strategies^186^ and are also undergoing sympatric speciation along a depth gradient in at least one location (Lake Masoko)^8^, where littoral and benthic *A. calliptera* ecomorphs have diverged in multiple characteristics, including body shape and trophic specialism, in approximately 1000 years^8^. Therefore, it is possible that morphological evaluations of more populations of *A. calliptera* will reveal further diversity, potentially providing greater insight into the role it has taken in generating the wider Lake Malawi haplochromine radiation and we would suggest increasing the sampling of *A. calliptera* populations to further investigate this.

A key part of macroevolutionary studies is the estimation of ancestral state of traits based on the morphology of their descendants^187^. This necessitates a comprehensive understanding of trait diversity across taxa, where such data is critical for the construction of models of morphological evolution, including estimating rates of phenotypic evolution. Since the genetic diversity of the Lake Malawi radiation was possibly seeded by multiple riverine species^5^, we sought to add specimens to the dataset that could enable the morphological reconstruction of the common ancestor of the Lake Malawi radiation. Therefore, we sampled two additional species of *Astatotilapia*. These included *Astatotilapia gigliolli* (n=2) and *Astatotilapia* sp. ‘Ruaha blue’ (n=2), native to the Great Ruaha River^182,183^. Construction of a mtDNA-based phylogeny initially placed *Astatotilapia* sp. ‘Ruaha blue’ as a sister taxa to the Lake Malawi radiation^182^. However, a phylogeny based on variation within whole-genome sequences has shown *A. gigliolli* and *A*. sp. ‘Ruaha blue’, sister taxa, form a sister clade with both the Lake Malawi and Lake Victoria radiations (see Fig. 1). This topology is likely the result of an ancestral hybridisation event with the ancestors of both lineages prior to their respective adaptive radiations^5^. Therefore, the addition of species from the Ruaha catchment, may therefore enable a more robust estimation of the ancestral phenotype of Lake Malawi cichlids.

### Deep Benthic and ‘*Utaka*’

We sampled deep-water benthic species from two genera; *Alticorpus* and *Lethrinops*^9,188^ (Table 1). *Alticorpus* is characterised by the presence of greatly enlarged cranial sensory openings and lateral line canals used to detect prey in the sediment. Deep-water benthic species are found below 50m, a ‘twilight’ zone with very little visible light. *Alticorpus macrocleithrum* (n=3) is found between 75m and 125m, with abundance peaking above 100m^189^, a depth similarly occupied by deep-water Lethrinops^190^, including *Lethrinops gossei* (n=1). Several species of *Lethrinops*, however, inhabit shallower water^4,169^. We sampled two species of shallow water *Lethrinops*, including *Lethrinops auritus* (n=2), and *Lethrinops albus* (n=2), both of which phylogenetically cluster within the ‘shallow benthic’ lineage (Fig. 1).

Our dataset^41^ also contains four species of zooplankton-feeding, shoaling cichlids which are commonly referred to as ‘utaka’. Utaka is primarily made up of species belonging to *Copadichromis*^191^, with a small number of species also belonging to *Mchenga* and *Nyassachromis*^192^. However, their placement within the utaka is disputed and they have not been considered in our species/genera counts (see Table 1). We sampled four species of *Copadichromis*: *Copadichromis likomae* (n=2), *Copadichromis quadrimaculatus* (n=2), *Copadichromis trimaculatus* (n=2, see Fig. 6) and *Copadichromis virginalis* (n=2). Utaka feed in the water column, and can be commonly found close to the shore^169^. *Copadichromis* are generally characterised by their relatively small, highly protrusible mouths, that they use to suck zooplankton into their mouths, as well as numerous long gill rakers which strain plankton from the water that enters their mouths as a result of their sucking feeding mechanism^169,193^.

Both the deep benthics and utaka are currently underrepresented within our dataset and future sampling should aim to add additional species of *Copadichromis*. In addition, sampling missing ‘deep benthic’ genera, such as *Aulonocara* and *Tramitichromis* should be prioritised for future sampling efforts. *Aulonocara stuartgranti* and *Aulonocara steveni* would be particularly interesting future additions and could offer interesting morphological comparisons with deeper living species. Moreover, given that *Lethrinops* is polyphyletic^190,194^, additional sampling of *Lethrinops* species could provide morphological data to support future systematic studies.

Whilst we were able to generate relatively good models for the specimens within this group (see Fig. 4 and Fig. 6), it is clear that some of the jaw did not resolve as well as in other specimens. Given the preferred ‘sucking’ zooplanktivore feeding mechanism of the species within *Copadichromis* it is possible that the jaw bones of these fish species are not particularly dense. This perhaps made it difficult to image these specimens using the same scanning procedure used for all the other remaining specimens. Therefore, in future, we would recommend care when segmenting craniofacial bones from the *Copadichromis* in this dataset and future sampling efforts should increase scanning exposure time and power to optimise specimen imaging.

### Processing and Segmentation Notes

The computer specifications we used for all processing steps (see Methodology) are hard to find on personal, or older machines and some users may find it difficult to work with some of our larger image stacks. To minimise memory usage during segmentation and speed up processing, cropped reconstructed stacks can be loaded in multiple increments (note that the Z-voxel size must be multiplied by said increment). We tested this and found that roughly comparable models could be generated, although it was clear that finer morphological detail was absent (data not shown). Therefore, where possible, the whole stack should be used when segmenting regions of interest. In addition, since these regions were manually segmented, many of the segmentation steps rely on the judgment of the individual segmenting and rendering the regions of interest. We found that segmenting from median-filtered reconstructed image stacks drastically lowered the quality of the rendered models (data not shown) and we suggest refraining from segmenting from a median-filtered image stack. In addition, we found that relatively low smoothing factors were best for rendering surfaces from segmented regions of interest. In Avizo Lite (v9.3.0), a smoothing factor between 0-10 (including rational intermediates) can be applied when rendering surfaces of segmented regions of interest. We rarely found it necessary to use a value above 3; indeed, all whole-body 3D models were smoothed with a factor of 2.5. Therefore, we suggest that regardless of the tool used to smooth and render segmented surfaces that smoothing be used conservatively. We also note that there are free, open access alternatives to Avizo Lite (v9.3.0) for the segmentation of 3D-image data, such as 3D-Slicer, which has a large and active community of users^195^ and Dragonfly which supports the use of deep learning to automatically segment 3D image data and offers non-commercial licenses, for academic use, free-of-charge. Both of these tools could be used in place of Avizo Lite (v9.3.0) for the segmentation steps outlined in Fig. 2.

## Supplementary information

Supplementary Table S2

Supplementary Table S1

Supplementary 3D Models

Supplementary Whole Body Images

## Data Availability

No custom code was used in the generation of this dataset.
