# Supplementary figures and images for: A whole-body micro-CT scan library that captures the skeletal diversity of Lake Malawi cichlid fishes (part 2 of 2)
Source: Sci Data. 2024 Sep 10;11:984. doi: 10.1038/s41597-024-03687-1 (PMC11387623; doi:10.1038/s41597-024-03687-1)

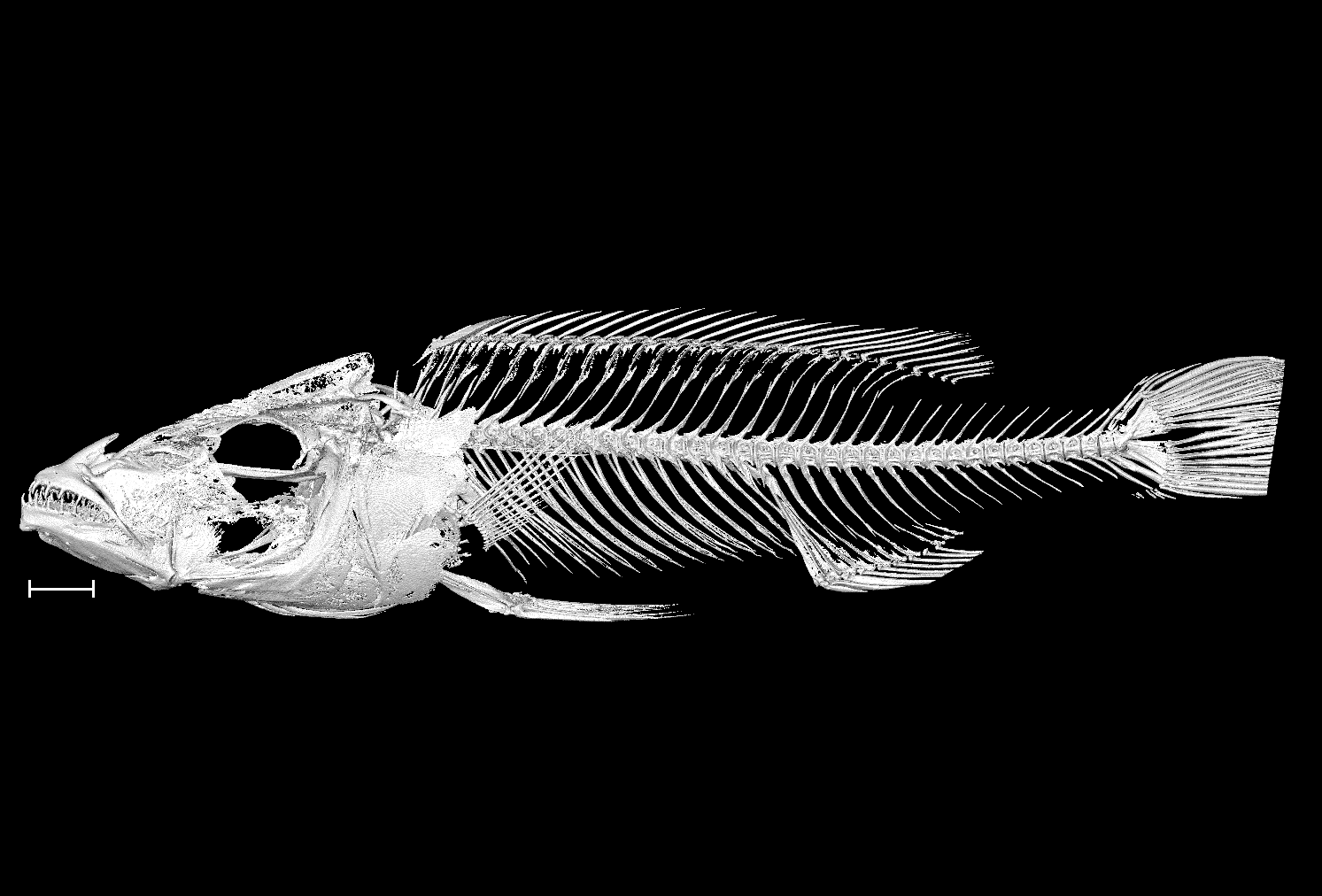

Supplement: Supplementary file 4 — Supplementary Whole Body Images [file 41597_2024_3687_MOESM4_ESM.zip › Whole_Body_Images/Rhamphochromis_sp_kingiri_large_UniBri_site1_8bit_a.tif]

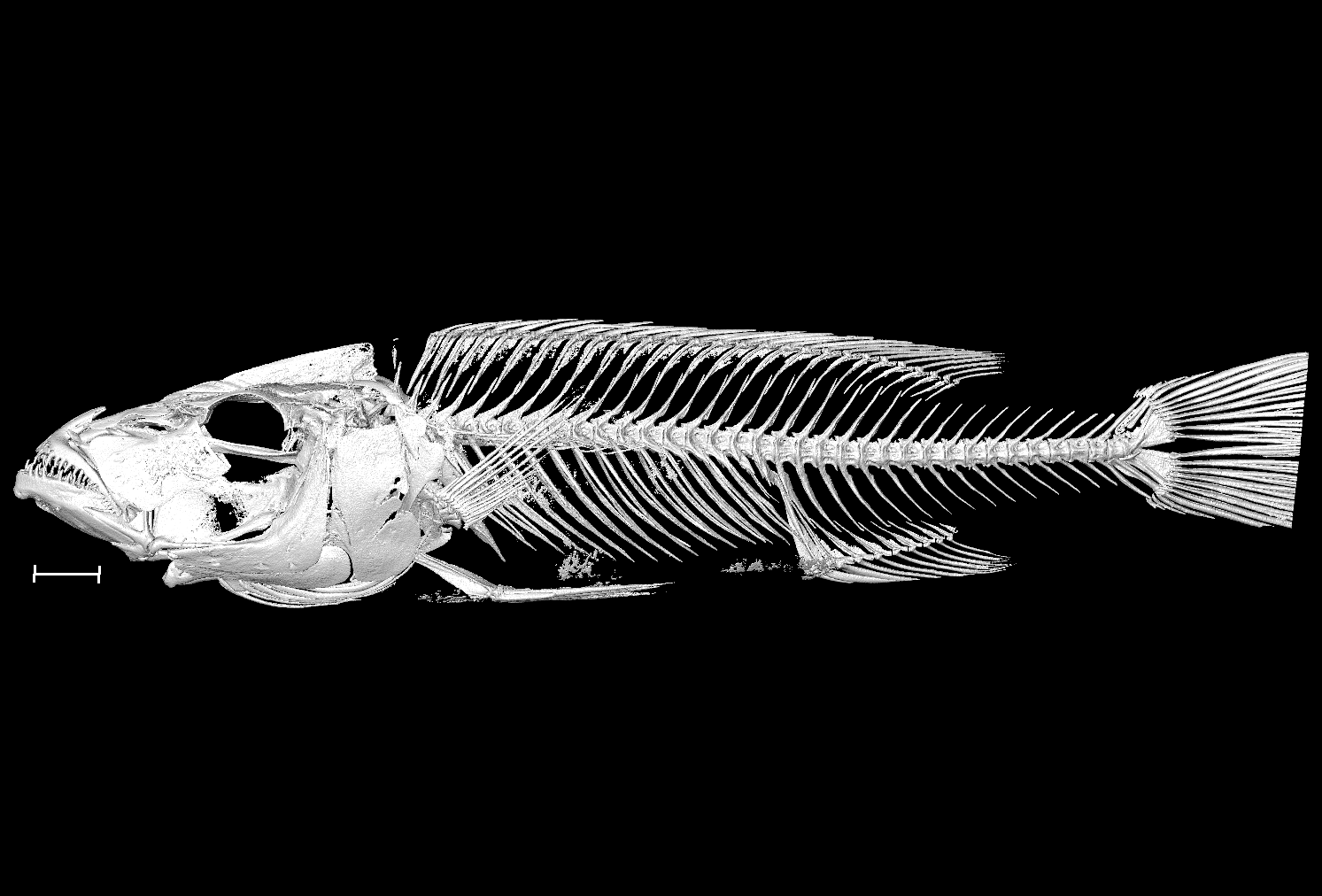

Supplement: Supplementary file 4 — Supplementary Whole Body Images [file 41597_2024_3687_MOESM4_ESM.zip › Whole_Body_Images/Rhamphochromis_sp_kingiri_large_UniBri_site1_8bit_b.tif]

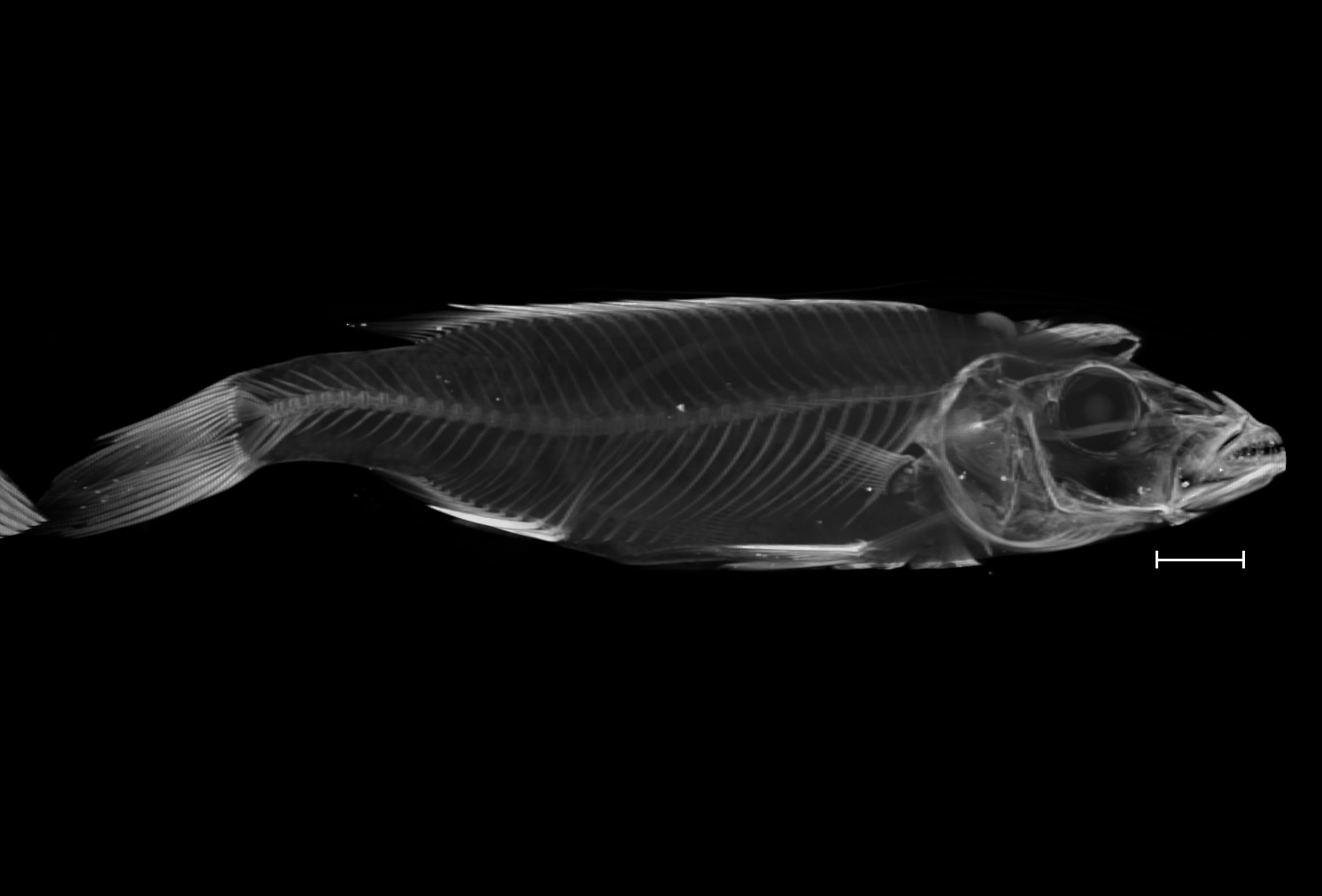

Supplement: Supplementary file 4 — Supplementary Whole Body Images [file 41597_2024_3687_MOESM4_ESM.zip › Whole_Body_Images/Rhamphochromis_sp_longiceps_blue_back_UniBri_RRC037_1_8bit.tif]

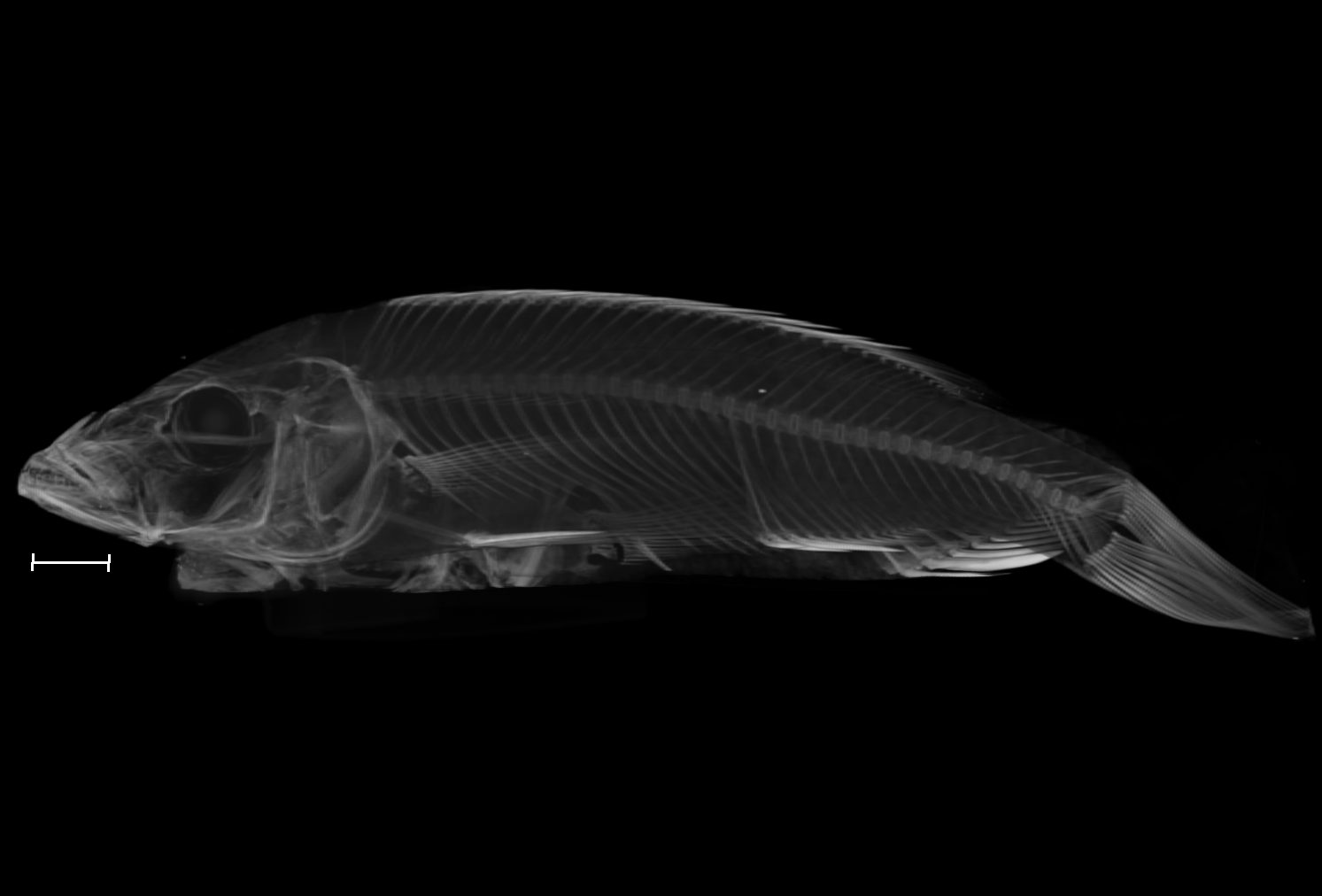

Supplement: Supplementary file 4 — Supplementary Whole Body Images [file 41597_2024_3687_MOESM4_ESM.zip › Whole_Body_Images/Rhamphochromis_sp_longiceps_blue_back_UniBri_RRC037_3_8bit.tif]

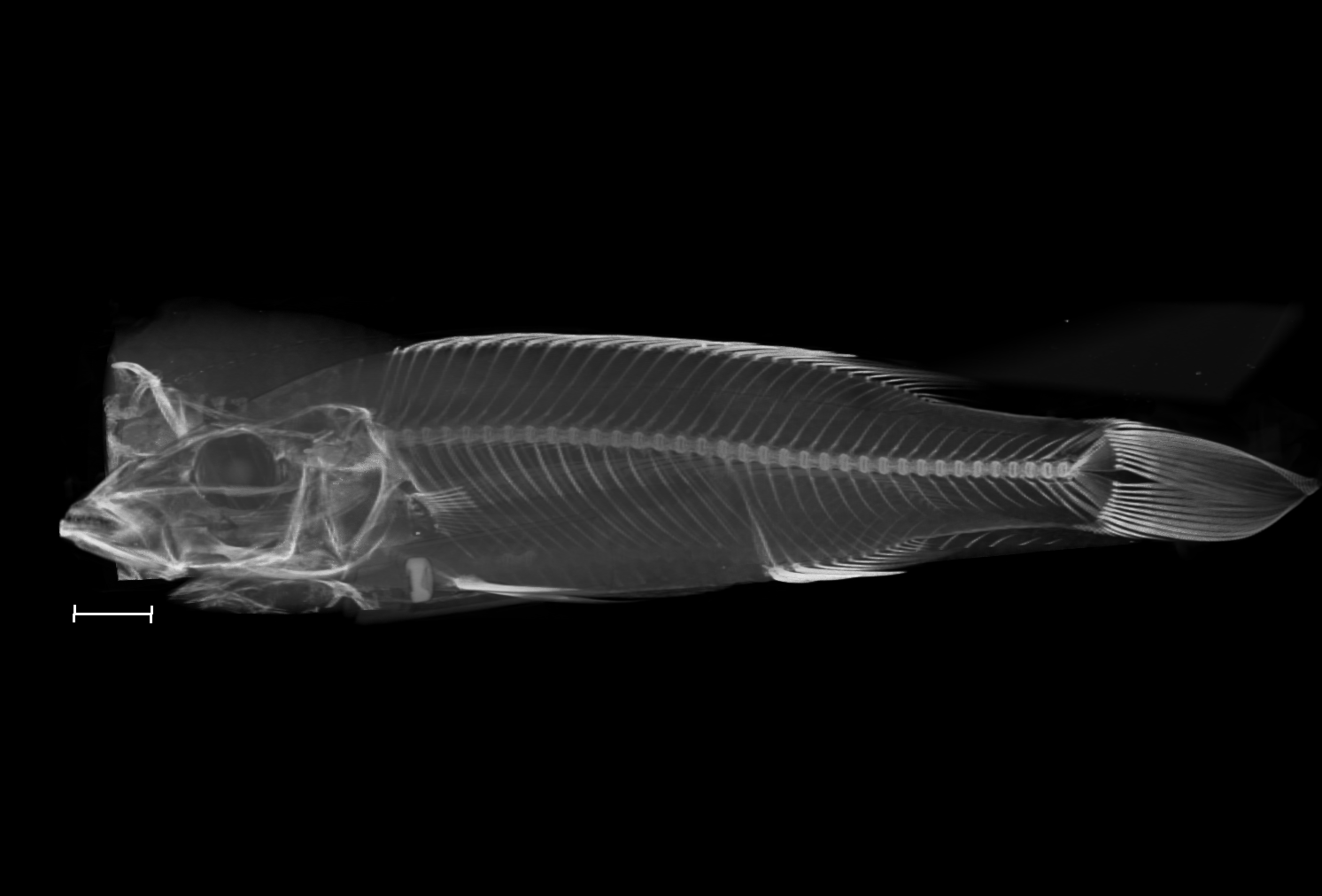

Supplement: Supplementary file 4 — Supplementary Whole Body Images [file 41597_2024_3687_MOESM4_ESM.zip › Whole_Body_Images/Rhamphochromis_sp_longiceps_grey_back_UniBri_RRC045_8bit_a.tif]

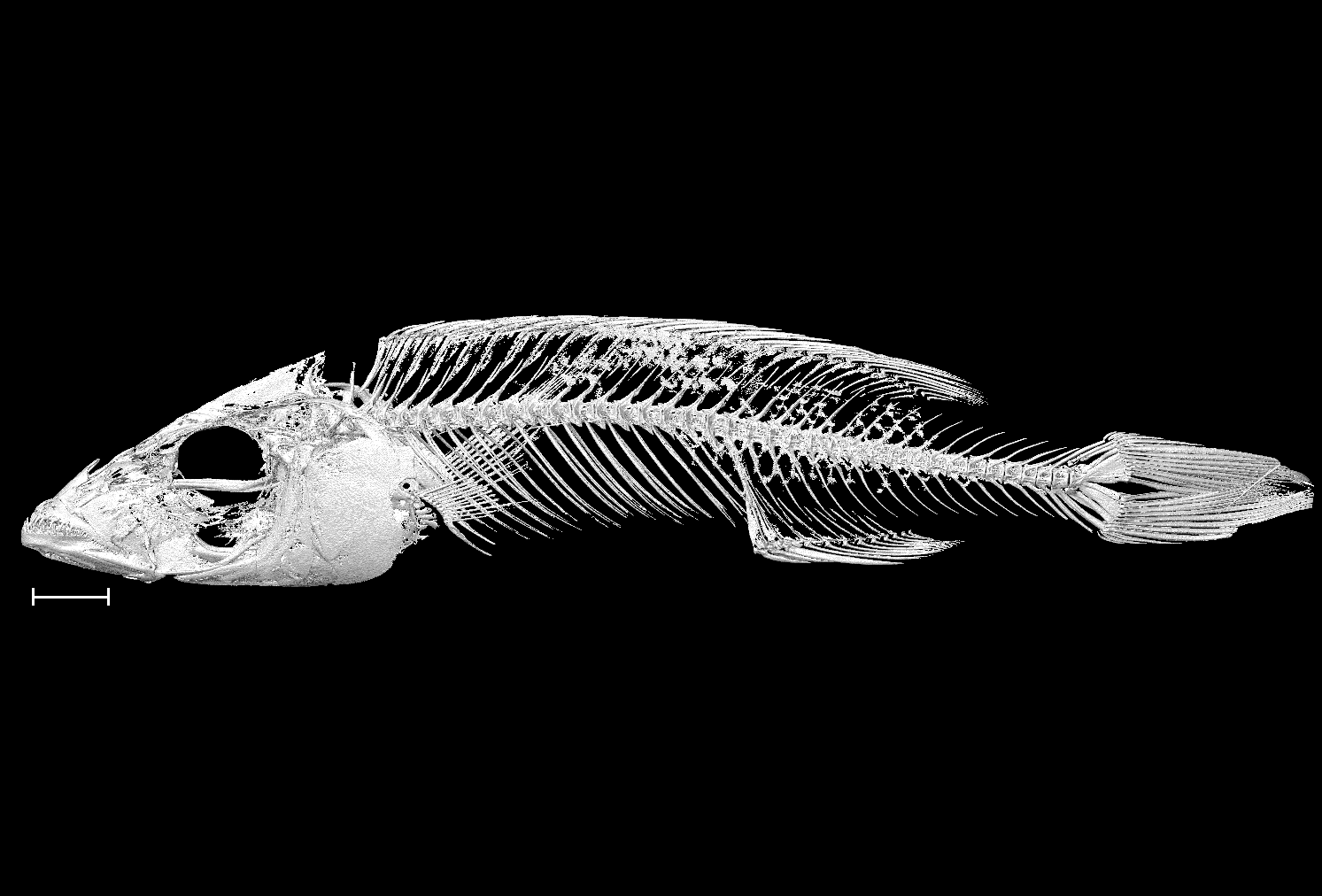

Supplement: Supplementary file 4 — Supplementary Whole Body Images [file 41597_2024_3687_MOESM4_ESM.zip › Whole_Body_Images/Rhamphochromis_sp_longiceps_grey_back_UniBri_RRC045_8bit_b.tif]

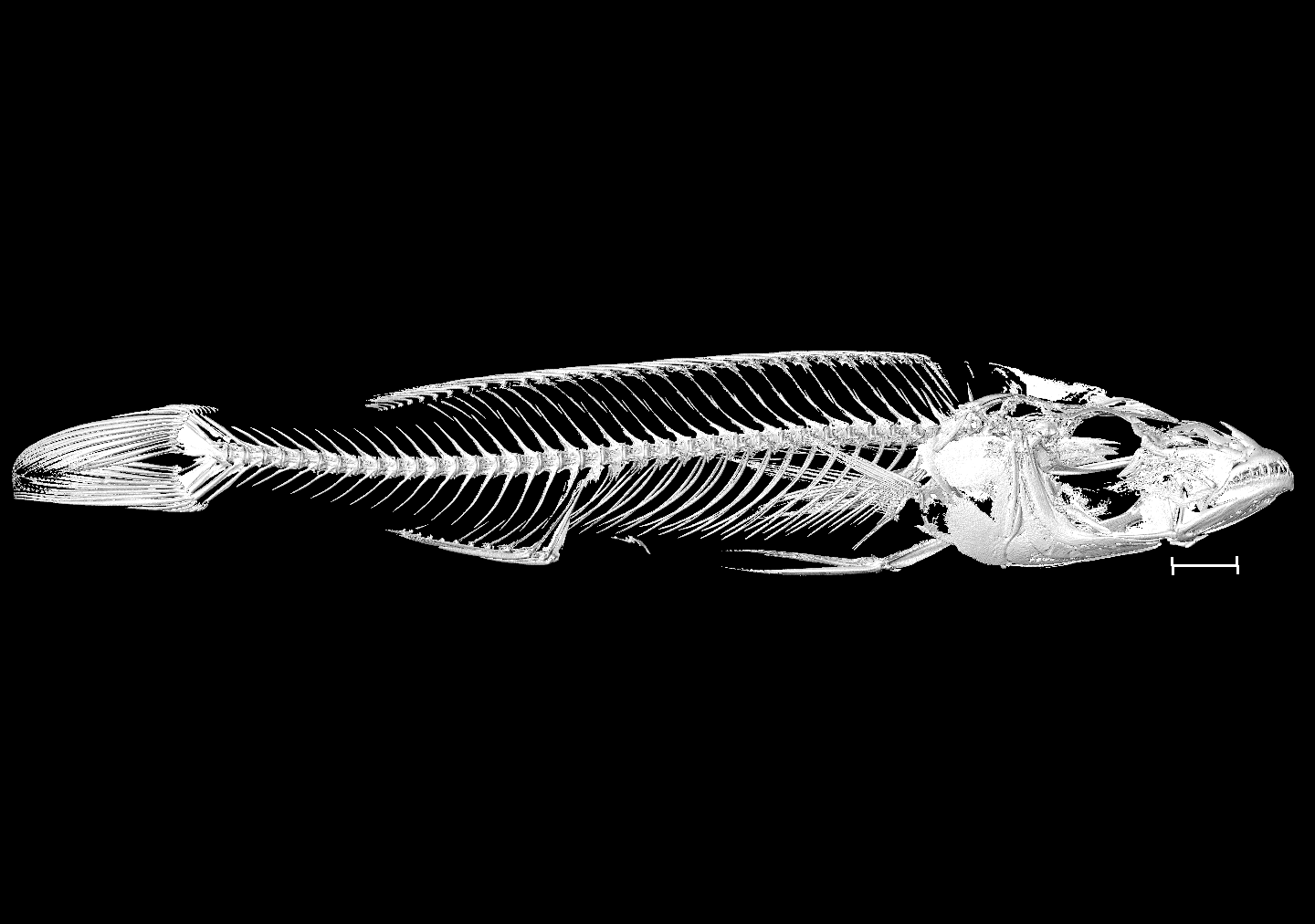

Supplement: Supplementary file 4 — Supplementary Whole Body Images [file 41597_2024_3687_MOESM4_ESM.zip › Whole_Body_Images/Rhamphochromis_sp_yellow_belly_UniBri_RRC049_8bit_a.tif]

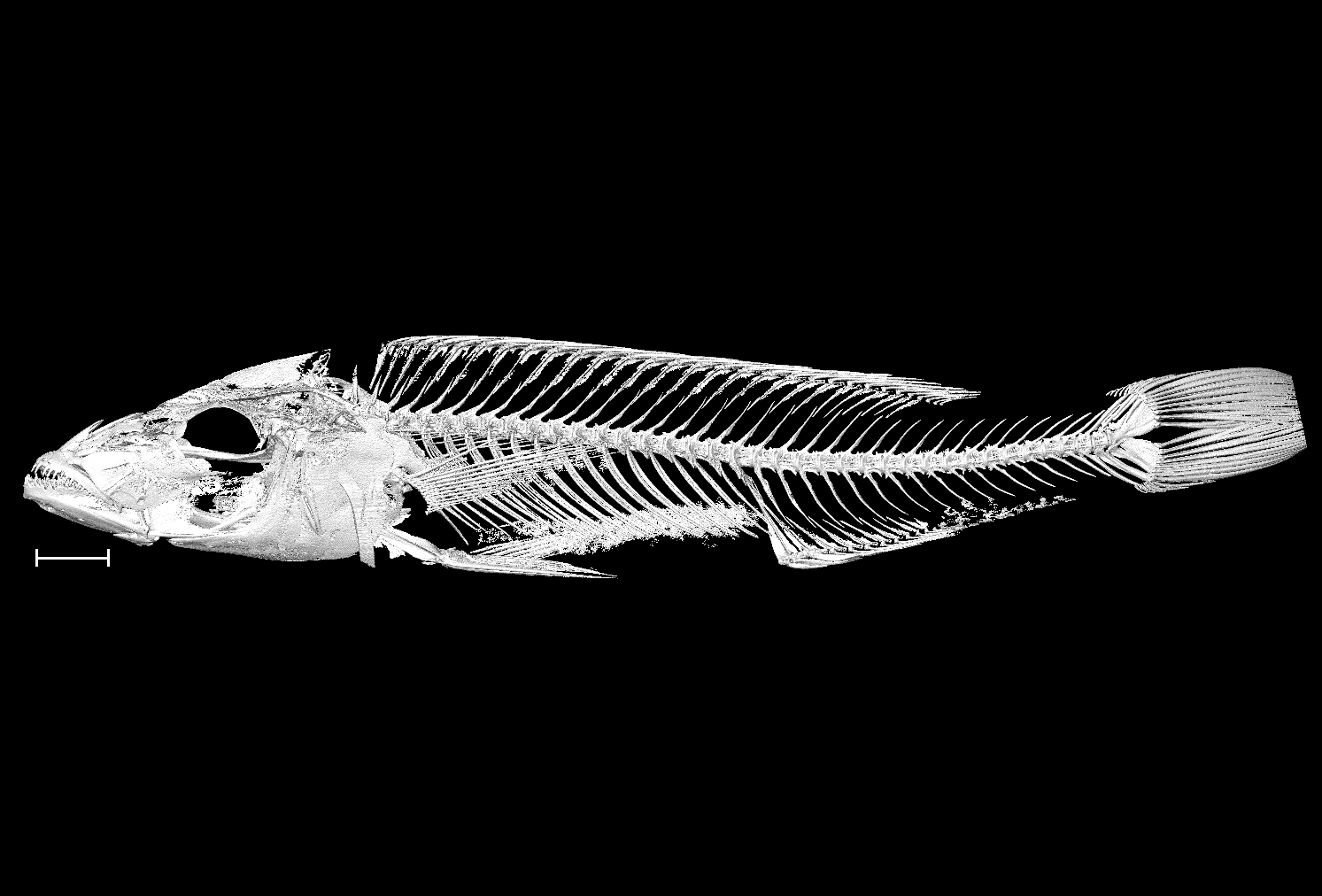

Supplement: Supplementary file 4 — Supplementary Whole Body Images [file 41597_2024_3687_MOESM4_ESM.zip › Whole_Body_Images/Rhamphochromis_sp_yellow_belly_UniBri_RRC049_8bit_b.tif]

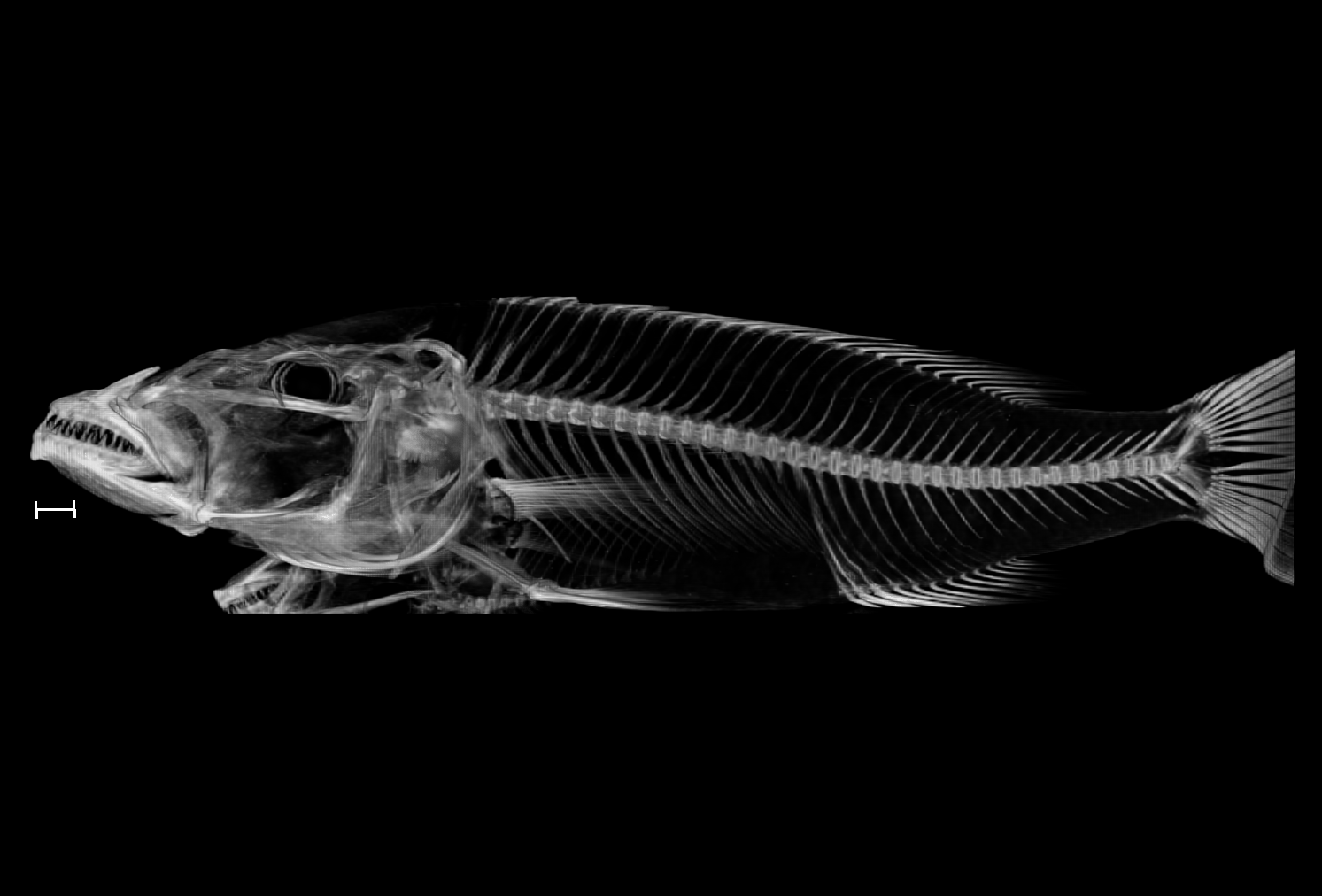

Supplement: Supplementary file 4 — Supplementary Whole Body Images [file 41597_2024_3687_MOESM4_ESM.zip › Whole_Body_Images/Rhamphochromis_woodi_NHMUK_1935_6_14_2185_2187_8bit_a.tif]

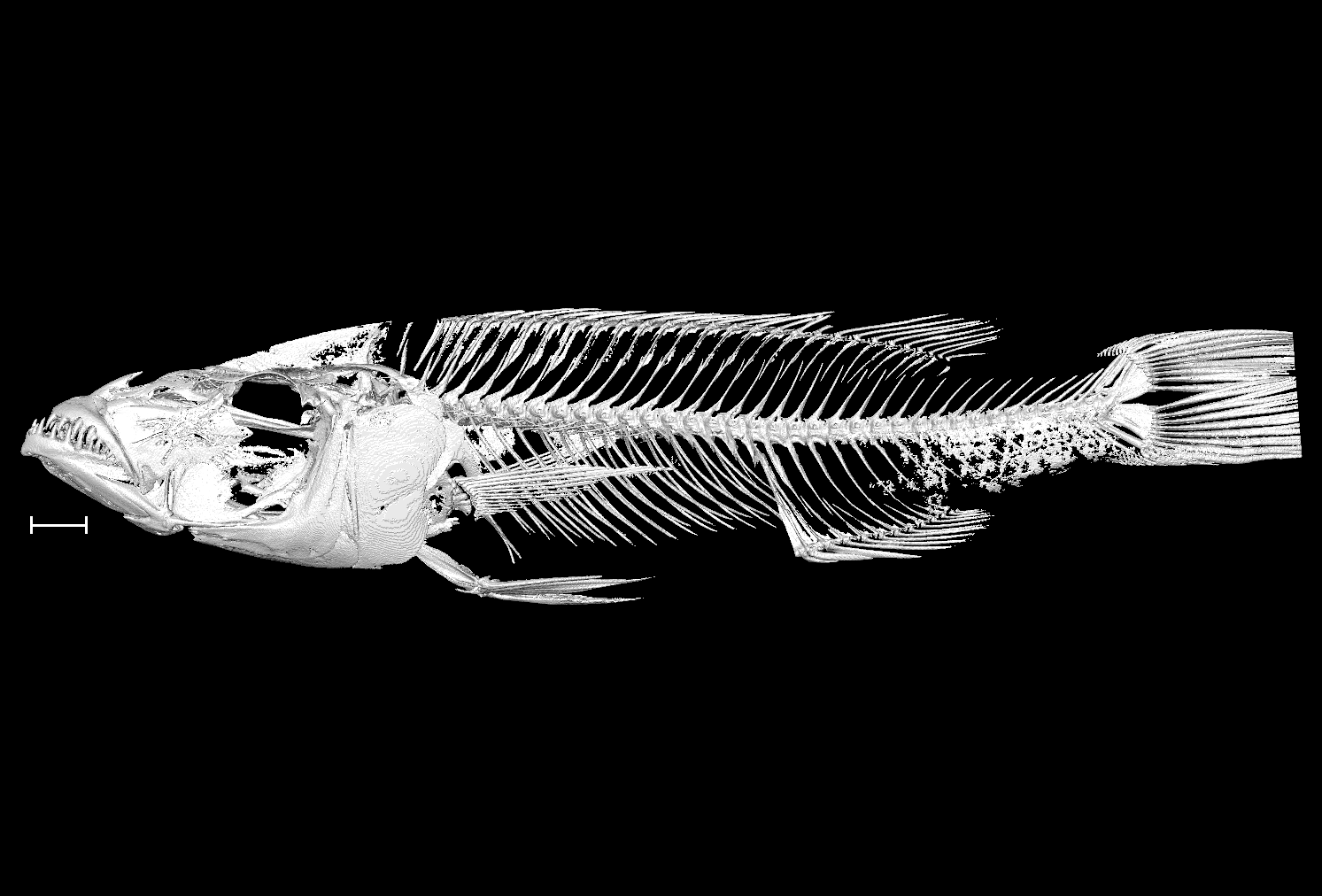

Supplement: Supplementary file 4 — Supplementary Whole Body Images [file 41597_2024_3687_MOESM4_ESM.zip › Whole_Body_Images/Rhamphochromis_woodi_NHMUK_1935_6_14_2185_2187_8bit_b.tif]

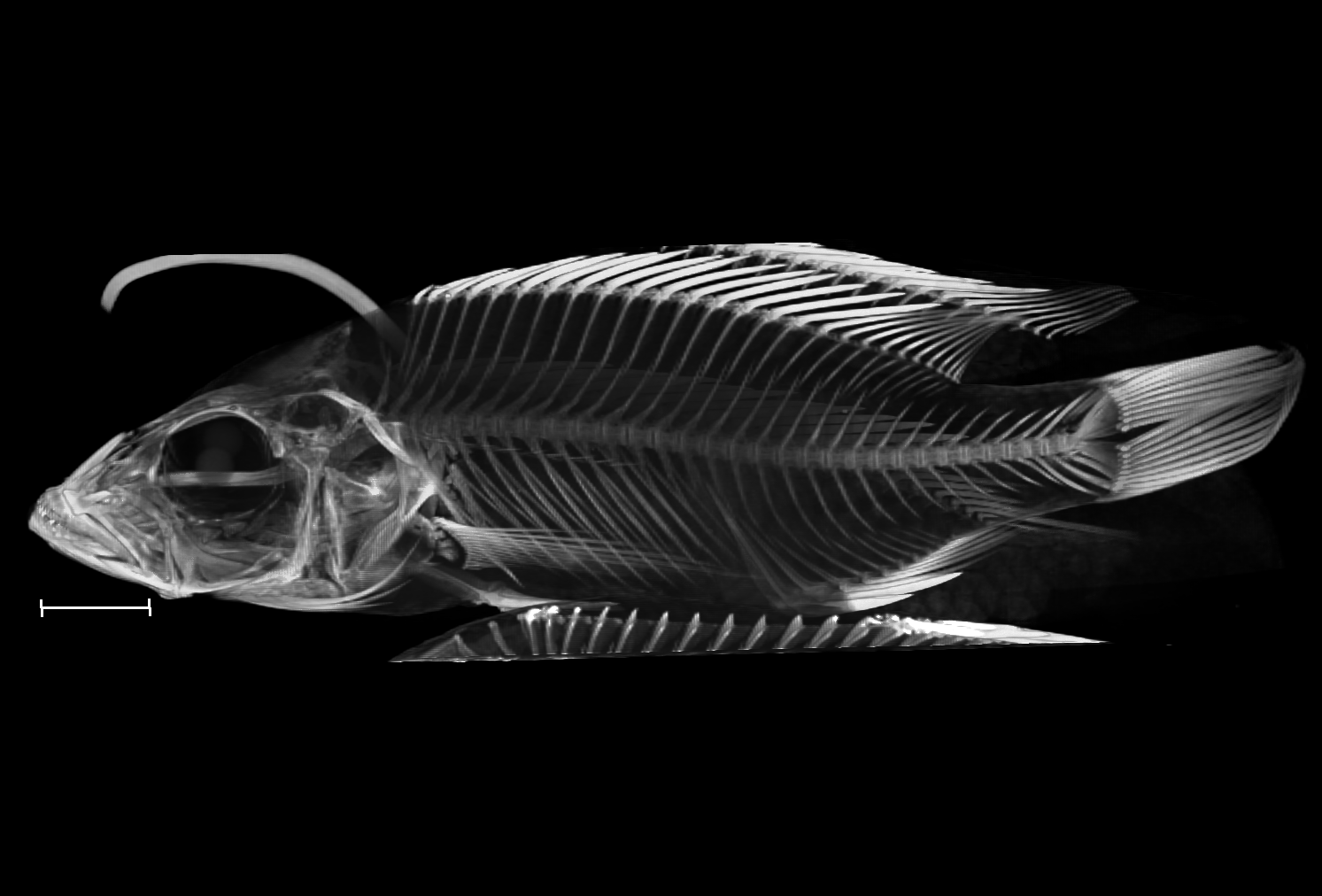

Supplement: Supplementary file 4 — Supplementary Whole Body Images [file 41597_2024_3687_MOESM4_ESM.zip › Whole_Body_Images/Stigmatochromis_macrorhynchos_UniBri_SG9_8bit.tif]

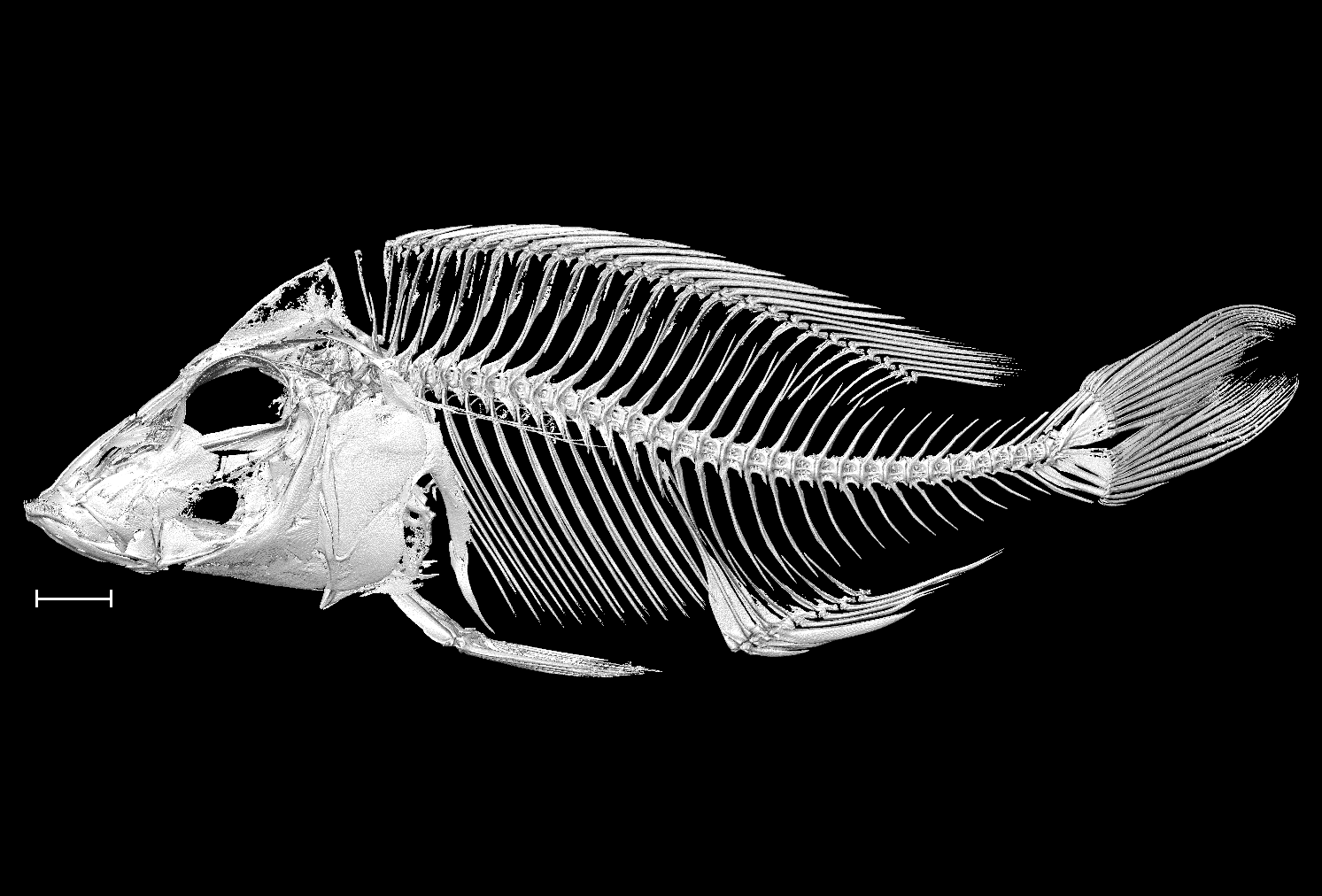

Supplement: Supplementary file 4 — Supplementary Whole Body Images [file 41597_2024_3687_MOESM4_ESM.zip › Whole_Body_Images/Taeniolethrinops_praeorbitalis_UniBri_31_9_14_8bit.tif]

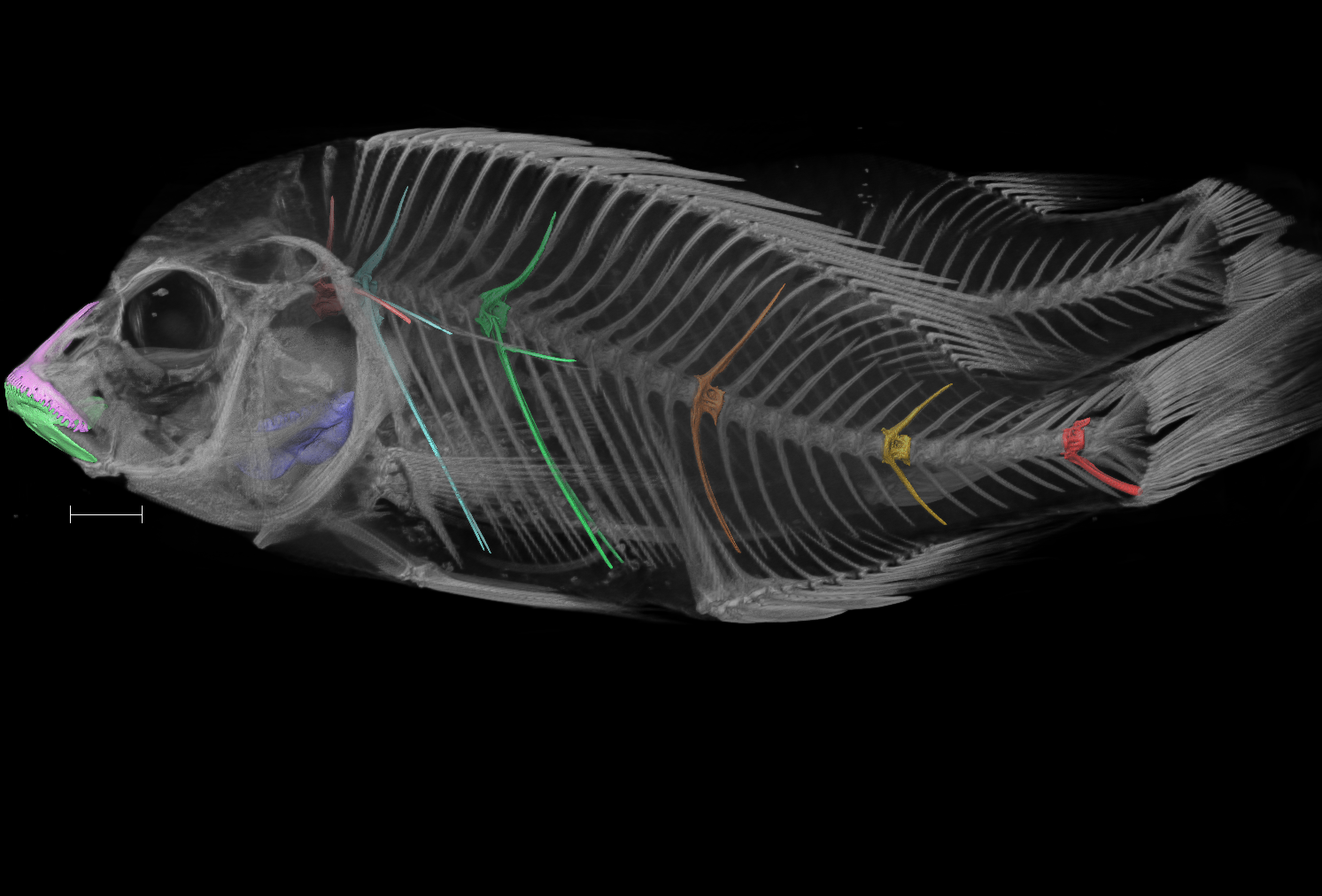

Supplement: Supplementary file 4 — Supplementary Whole Body Images [file 41597_2024_3687_MOESM4_ESM.zip › Whole_Body_Images/Trematocranus_placodon_UniBri_220_8bit.tiff]

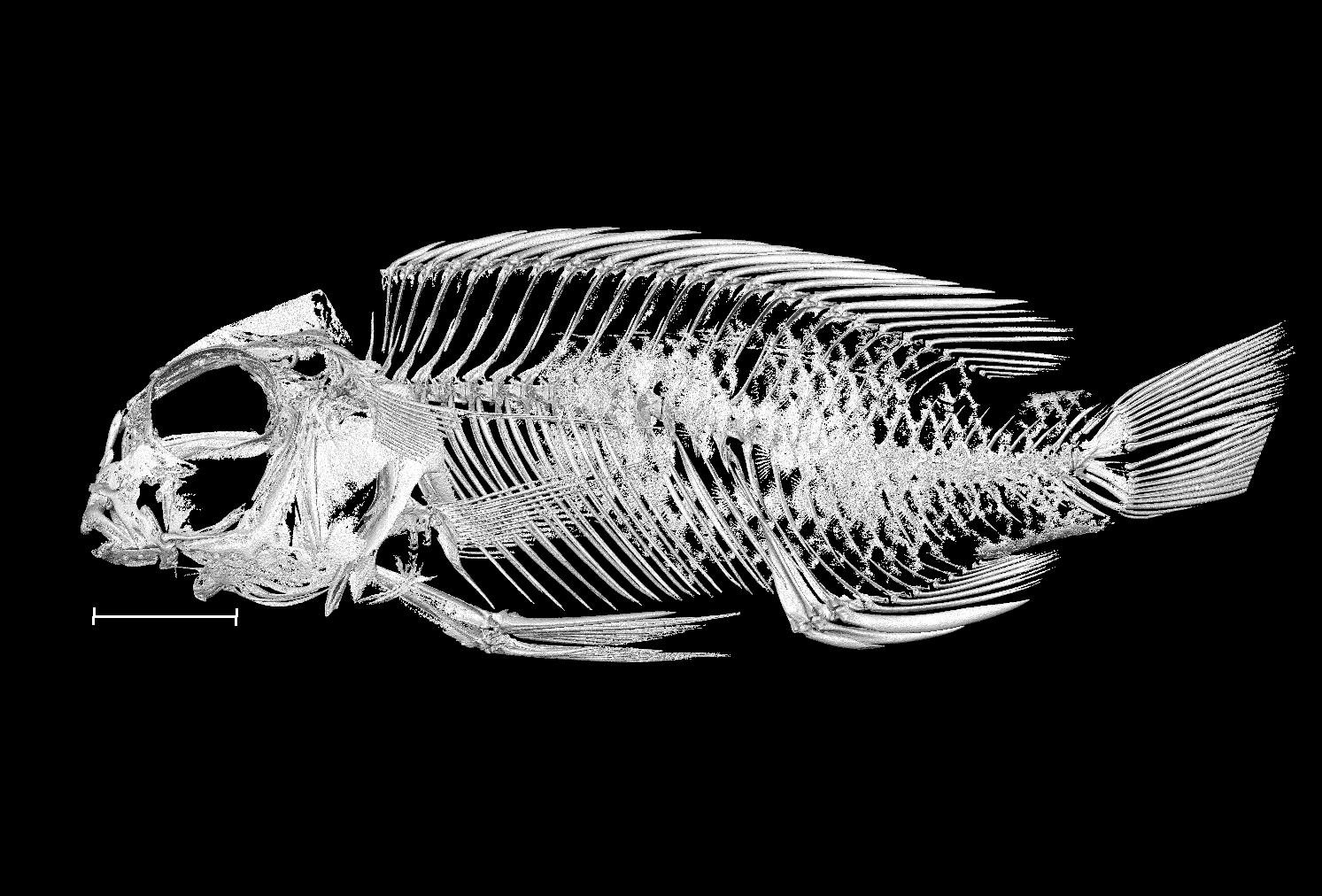

Supplement: Supplementary file 4 — Supplementary Whole Body Images [file 41597_2024_3687_MOESM4_ESM.zip › Whole_Body_Images/Tropheops_tropheops_NHMUK_2012_1_17_18_25_8bit_a.tif]

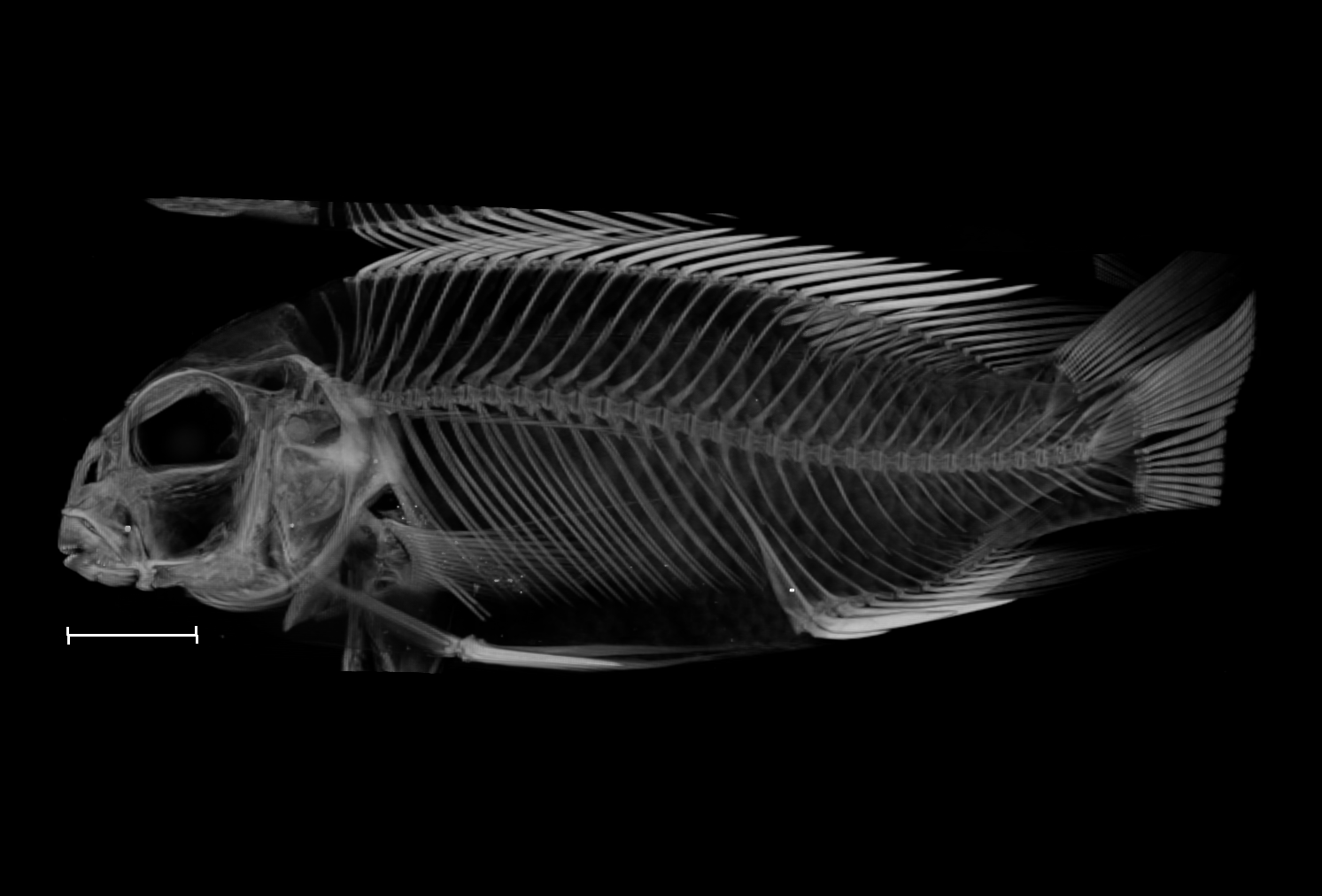

Supplement: Supplementary file 4 — Supplementary Whole Body Images [file 41597_2024_3687_MOESM4_ESM.zip › Whole_Body_Images/Tropheops_tropheops_NHMUK_2012_1_17_18_25_8bit_b.tif]

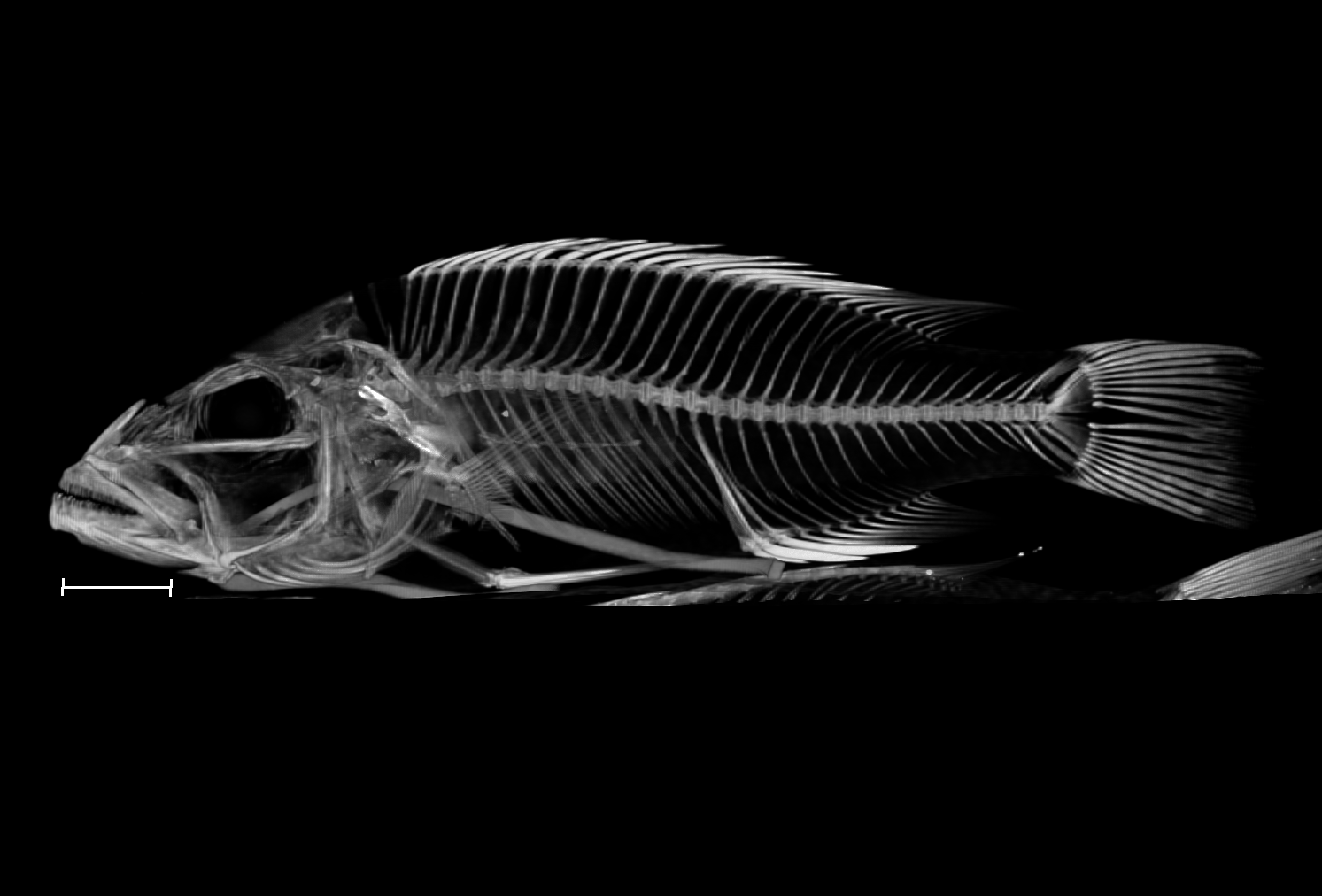

Supplement: Supplementary file 4 — Supplementary Whole Body Images [file 41597_2024_3687_MOESM4_ESM.zip › Whole_Body_Images/Tyrannochromis_macrostoma_UniBri_201_8bit.tif]
